# Exploring the relationship between oculomotor preparation and gaze-cued covert shifts in attention

**DOI:** 10.1167/jov.23.3.18

**Published:** 2023-03-30

**Authors:** Samantha Parker, Richard Ramsey

**Affiliations:** 1 School of Psychological Sciences, Macquarie University, Sydney, New South Wales, Australia

**Keywords:** gaze-cueing, attention, saccade, evidence accumulation modelling, eye movements

## Abstract

Eye gaze plays dual perceptual and social roles in everyday life. Gaze allows us to select information, while also indicating to others where we are attending. There are situations, however, where revealing the locus of our attention is not adaptive, such as when playing competitive sports or confronting an aggressor. It is in these circumstances that covert shifts in attention are assumed to play an essential role. Despite this assumption, few studies have explored the relationship between covert shifts in attention and eye movements within social contexts. In the present study, we explore this relationship using the saccadic dual-task in combination with the gaze-cueing paradigm. Across two experiments, participants prepared an eye movement or fixated centrally. At the same time, spatial attention was cued with a social (gaze) or non-social (arrow) cue. We used an evidence accumulation model to quantify the contributions of both spatial attention and eye movement preparation to performance on a Landolt gap detection task. Importantly, this computational approach allowed us to extract a measure of performance that could unambiguously compare covert and overt orienting in social and non-social cueing tasks for the first time. Our results revealed that covert and overt orienting make separable contributions to perception during gaze-cueing, and that the relationship between these two types of orienting was similar for both social and non-social cueing. Therefore, our results suggest that covert and overt shifts in attention may be mediated by independent underlying mechanisms that are invariant to social context.

## Introduction

Eye gaze is a powerful social tool, which serves multiple functions. Gaze enables features in the environment to be processed in depth, while also signaling our intentions to others (Baron-Cohen, Wheelwright, & Jolliffe, 1997; Risko, Richardson, & Kingstone, 2016). Consequently, it is unsurprising that the ability of another human's gaze to direct attention covertly (without eye movements) and overtly (with eye movements) has been the subject of intense investigation over the past three decades (Frischen, Bayliss, & Tipper, 2007). Although much is known about the effects of another's gaze direction on these orienting mechanisms separately, few studies have investigated the relationship between covert and overt shifts in attention during gaze-cueing. This is despite intense debate and investigation of this relationship in the general attention literature more broadly (Casteau & Smith, 2019; Hunt, Reuther, Hilchey, & Klein, 2019a; Smith & Schenk, 2012). Therefore, in the current study, we combine computational modeling with a gaze-cueing paradigm to explore the relationship between eye movements and attention directed by social cues. The results shed new light on the relationship between covert and overt orienting during social and non-social cueing.

The gaze-cueing paradigm has been used extensively to investigate the extent to which attention is oriented to the gaze direction of others (Driver, Davis, Ricciardelli, Kidd, Maxwell, & Baron-Cohen, 1999; Friesen & Kingstone, 1998). In this variant of a traditional Posner cueing paradigm (Posner, 1980), participants are presented with a schematic or photograph of a face at fixation. The gaze direction of the cue is then aligned toward the location of an upcoming peripheral target (valid cue condition) or away from it (invalid cue condition). In the classic version of this task, participants are required to maintain central fixation while manual responses are measured. Studies typically report that discrimination and detection of a target are faster and more accurate when preceded by a valid gaze cue relative to an invalid gaze cue. As the participants’ eyes remain at fixation throughout the task, the difference in performance between valid and invalid conditions is typically attributed to a covert shift in spatial attention. Gaze-cues have also been found to have a similar influence on overt shifts in attention. That is, eye movements, or saccades, are initiated faster, and are less likely to land in the wrong position when preceded by a valid gaze-cue relative to an invalid gaze-cue (Dalmaso, Castelli, & Galfano, 2020; Itier, Villate, & Ryan, 2007; Kuhn & Benson, 2007; Kuhn & Kingstone, 2009; Ricciardelli, Bricolo, Aglioti, & Chelazzi, 2002).

Although these prior studies have improved our understanding of gaze-cued shifts of covert and overt orienting separately, very little research has experimentally investigated the relationship between these mechanisms during gaze-cueing. Consequently, it remains unclear how these two complementary forms of orienting interact when attention is directed by a social cue. The lack of investigation of this question is surprising for two reasons: (1) there is a growing number of studies that suggest both covert and overt orienting play a role in guiding and facilitating attention during social interactions (Kuhn, Tatler, & Cole, 2009; Kuhn, Tatler, Findlay, & Cole, 2008; Kuhn, Teszka, Tenaw, & Kingstone, 2016; Laidlaw, Rothwell, & Kingstone, 2016); and (2) the relationship between spatial attention and eye movements has been the subject of intense investigation within the general attention literature more broadly for over four decades (Klein, 1980; Posner, 1980; Rizzolatti, Riggio, Dascola, & Umilta, 1987).

One particularly pervasive question concerns the extent to which these two types of orienting are obligatorily coupled or dissociable (Casteau & Smith, 2019; Hunt et al., 2019a; Smith & Schenk, 2012). Although early studies suggested that spatial cues could not shift the locus of attention away from the goal of an upcoming eye movement (Hoffman & Subramaniam, 1995; Rizzolatti et al., 1987), more recent research has called into question this conclusion (Casteau & Smith, 2018; Castet, Jeanjean, Montagnini, Laugier, & Masson, 2006; Montagnini & Castet, 2007; Parker, Heathcote, & Finkbeiner, 2020a; Parker, Heathcote, & Finkbeiner, 2020b; Parker, Heathcote, & Finkbeiner, 2021; Smith & Schenk, 2012). There is now growing acceptance within the general attention literature that each type of orienting is, at least partly, dissociable and likely mediated by independent underlying mechanisms (Casteau & Smith, 2019; Hunt et al., 2019a; Klein, 2020; Li, Pan, & Carrasco, 2021).

Recent research on social attention in ecologically valid scenarios suggests that spatial attention and eye movements may both play a role during social interactions (Kuhn et al., 2008; Kuhn et al., 2009; Laidlaw et al., 2016). Laidlaw and colleagues (2016), for example, found that people tended to use covert attention to monitor their social environment to determine whether subsequent eye contact was appropriate. On the other hand, Kuhn and colleagues (2008; Kuhn et al., 2009; Kuhn et al., 2016), monitored the covert and overt attention of observers viewing live and recorded magic tricks. The authors reported that although overt attention could be modulated by knowledge of the trick, covert attention could not. When taken together, these studies suggest that covert and overt orienting in social environments play complementary yet distinct roles. In addition, Morgan, Ball, and Smith (2014) supported and extended this conclusion using an eye abduction paradigm. The eye abduction paradigm involves artificially restricting eye movements in healthy participants by rotating the visual display to an angle of 40 degrees from one eye (Craighero, Nascimben, & Fadiga, 2004). By virtue of this rotation, it is thought that eye movements cannot be prepared or executed toward the restricted hemifield. The authors reported no cueing effects in the restricted hemifield for peripheral or arrow cues, however, the gaze-cueing effect remained intact. These results led the authors to suggest gaze-cueing did not depend upon oculomotor preparation, and that socially cued attention was likely mediated by a unique underlying mechanism to that associated with non-socially cued attention.

Whereas Morgan and colleagues (2014) suggest that there may be a dissociation between covert and overt orienting to social information, this study suffers from two limitations, which makes firm conclusions difficult to make. First, the experimental design used by Morgan and colleagues (2014) involved restricting eye movements, which means it remains to be seen if covert and overt orienting can independently contribute to performance in the same gaze-cueing task when participant's eye movements are not artificially restricted. Second, Morgan and colleagues (2014) analyzed reaction time (RT) and accuracy separately and made comparisons between task blocks. In such designs, it is not possible to disentangle the effects of response caution, which can vary across blocked task conditions, from true differences in the attention effect on performance (Donkin, Averell, Brown, & Heathcote, 2009). Imagine, for example, that participants displayed more cautious responding to gaze cues in one block, such that responses were slower but more accurate, and less cautious responding to peripheral cues in a separate block, such that decisions were faster but less accurate. It is difficult to disentangle in a separate analysis of accuracy and RT whether these differences in performance are due to differences in response caution, or due to the operation of the cue. Consequently, it is unclear to what extent the relationship between covertly oriented spatial attention and saccade preparation varies by cue type.

**Figure 1.**
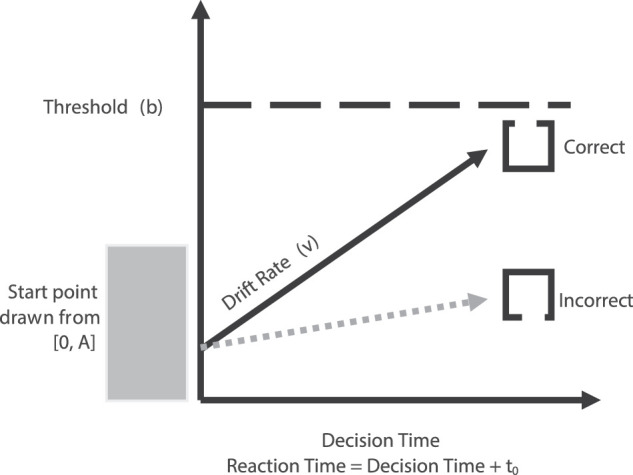
Schematic of LBA model applied to a single trial of the study. Note that the participant's task was to detect the gap location of a Landolt square. In this example, the stimulus presented had a gap in the top edge of the square. Quality of evidence accumulation is measured by taking the difference between the drift rate (*v*) for the correct response (e.g. that the gap occurred in the top edge of the square, black solid line) and the incorrect response (e.g. that the gap occurred in the bottom edge of the square, dotted grey line). A decision will be made once evidence in favor of one decision crosses the threshold (*b;* dashed black line).

The current study addresses these limitations in two ways. First, we used an experimental design – the saccadic dual task – that can independently manipulate eye movements and gaze-cues in order to investigate the relationship between covert shifts in spatial attention and overt shifts in attention that are associated with preparing an eye movement (Castet et al., 2006; Deubel, 2008; Hoffman & Subramaniam, 1995; Montagnini & Castet, 2007). Second, we analyzed the data using an evidence accumulation computational model that allows comparisons to be made across both blocked task conditions and between subjects’ conditions by providing a principled way of combining accuracy and the distribution of RTs for correct and error responses (Brown & Heathcote, 2008; Donkin, Brown, & Heathcote, 2011; see Figure 1). Importantly for our purposes, this parameterization allows differences in response caution to be quantified and separated from differences due to task difficulty. The benefit of doing so is that we can draw inferences about orienting without contamination from strategic differences that can occur across blocked task conditions.

Combining the saccadic dual task with evidence accumulation modeling has recently been used to study the relationship between covert and overt orienting in non-social settings (Parker et al., 2020a; Parker et al., 2020b; Parker et al., 2021). Across these studies, the authors reported a separate and dissociable contribution of both covert orienting and saccade preparation, the magnitude of which differed in size. The influence of preparing an eye movement on performance was greater than that of a sudden onset peripheral cue. Furthermore, when the spatial acuity demands of the perceptual discrimination task were increased the authors reported that the magnitude of the saccade congruency effect selectively increased (Parker et al., 2021). When taken together these results led the authors to conclude that covertly oriented spatial attention and eye movement preparation made a quantitatively and qualitatively distinct contribution to perception and that covert and overt orienting were likely mediated by distinct underlying mechanisms.

The aim of the present study was to build on the work by Parker and colleagues (2020a; Parker et al., 2020b; Parker et al., 2021) and use evidence accumulation modelling to explore the relationship between covertly oriented spatial attention and oculomotor preparation during social and non-social cueing tasks. Across two experiments, a cueing paradigm was used to direct spatial attention covertly while eye movements were prepared or remained at fixation. The spatial cue could be a social gaze-cue or a non-social arrow cue (Kuhn & Kingstone, 2009; Morgan et al., 2014). We took as our main dependent measure the drift rate parameter of the Linear Ballistic Accumulator (Brown & Heathcote, 2008), as this parameter can capture both the quality and amount of information accumulating about a decision (Lewandowsky & Oberauer, 2018). Given these characteristics drift rates provide a measure by which to compare the contributions of covert and overt orienting across tasks and cue types that is not contaminated by differences that can occur across blocked or between subject conditions. We preregistered three hypotheses about the relationship between each type of orienting during social cueing. First, if covertly oriented spatial attention and saccade preparation during gaze-cueing are dissociable (Kuhn et al., 2008; Kuhn et al., 2016; Laidlaw et al., 2016; Morgan et al., 2014), there will be a distinct contribution of both saccade congruency and cue validity to performance on dual-task trials. That is, we predict an effect of both saccade congruency and cue validity on performance. Furthermore, consistent with previous studies (Parker et al., 2020a; Parker et al., 2020b; Parker et al., 2021), we predict that the magnitude of these effects will differ such that the magnitude of the saccade congruency effect will be greater than the cue validity effect. Second, if both types of orienting are mediated by independent underlying mechanisms, the magnitude of the gaze cueing effect (difference between a valid and invalid gaze cued trial in drift rate) will not vary as a function of eye movement preparation or fixation (Parker et al., 2020a; Parker et al., 2020b; Parker et al., 2021). Third, if similar underlying mechanisms mediate the relationship between each type of orienting during social and non-social cueing, we predict that the same pattern of dissociation between covertly oriented spatial attention and saccade preparation evident for gaze-cues will also be present for nonpredictive centrally presented arrow cues. That is, for both cue types, there will be a main effect of saccade congruency, cue validity, and no interaction between the two. We have no specific predictions about how the magnitude of the main effects may differ by cue type.

## Method

In experiment 1a spatial attention was directed covertly by a gaze-cue presented at fixation, whereas in experiment 1b spatial attention was directed covertly by a centrally presented arrow. Both cue types were non-predictive of target location. Except for cue type, all other aspects of the task were identical across experiments and we therefore present the methods and results of both experiments together. Across both experiments, participants were required to identify the location of a small gap located in either the top or bottom edge of a Landolt square (originally adapted from Montagna, Pestilli, & Carrasco, 2009).

Each experiment consisted of two different tasks: (a) the *fixation* task where participants were required to maintain central fixation and eye movements were not monitored and (b) the *dual task* in which participants were required to complete the discrimination task and make an eye movement to an instructed location at the same time, while their eye movements were monitored. As previous studies have found that the size of the cueing effect is similar when participants are required to maintain fixation regardless of whether their eye movements are monitored or not (Parker et al., 2021), we did not monitor eye movements in the fixation task.

### Pre-registration and open science statement

The research question, design, hypotheses, planned analysis, sample size, and exclusion criteria for both experiments 1a and 1b were preregistered (https://osf.io/dw4c9). In addition, all raw data, stimuli, and analysis code for each experiment have been made available on the open science framework (see https://osf.io/fe9ds/).

We deviated from our preregistered analysis in one way. Originally, we had intended to use maximum likelihood estimation (MLE) to fit our evidence accumulation model to accuracy and reaction time data. We then planned to analyze the resulting estimates of drift rate difference (difference in drift rate between the true and false accumulators) and threshold using Bayesian multilevel modelling (McElreath, 2020). Instead, we fit the linear ballistic accumulator (LBA) model using a hierarchical Bayesian approach with the Dynamic Models of Choice (DMC) software in R (Heathcote, Lin, Reynolds, Strickland, Gretton, & Matzke, 2019). As a result, rather than submitting the resulting estimates to multilevel modeling, we were able to directly inspect the posterior distributions of parameters estimates. Importantly, all other aspects of the analysis were completed as outlined in the pre-registration, including the main dependent variable of interest (drift rate difference).

This deviation from preregistration was necessary because we were unable to fit the models using MLE because we did not have access to a required server. However, there are several benefits of the Bayesian approach to modeling. First, unlike with the MLE method, the Bayesian estimation technique allowed us to quantify the uncertainty that surrounds parameter estimates, an important factor for sound statistical inference (Evans & Wagenmakers, 2020; Heathcote et al., 2019; Lin & Stickland, 2020). Second, the software that we used to fit these Bayesian models and estimate the parameters is open source and freely available to run in the R computing language and is accompanied by a set of extensive tutorials on how to build such models (Heathcote et al., 2019).

### Participants

All participants were from Macquarie University and participated in return for course credit or remuneration. Participants all had normal or corrected to normal vision. All procedures were approved by the local ethics committee and participants provided informed consent before participating.

**S**ample size was determined in advance with regard to practical constraints, including task duration (approximately 2 hours per participant) and available resources. Specifically, we set out to collect the maximum amount of data that we could reasonably collect per participant in one testing session, and the maximum number of participants that the available resources would allow, in terms of researcher time. Ninety-six participants took part in the study. Forty-eight participants took part in experiment 1a (14 men and 34 women) with 24 participants in the fixation condition and 24 in the dual task condition. Ages ranged from 18 to 34 years (*M* = 19.90, *SD* = 2.66). Fourteen participants were replaced, five for failing to reach above chance accuracy (>55% overall accuracy) on the Landolt gap detection task and nine for failing to make the correct eye movement on over 75% of trials. Forty-eight participants took part in experiment 1b (17 men and 31 women), again 24 per task condition. Ages ranged from 18 to 25 years old (*M* = 19.49, *SD* = 1.79). Thirteen participants were replaced in experiment 1b, six for failing to achieve above chance accuracy and a further seven for failing to make the correct eye movements on a sufficient number of trials.

### Trial structure

**Figure 2.**
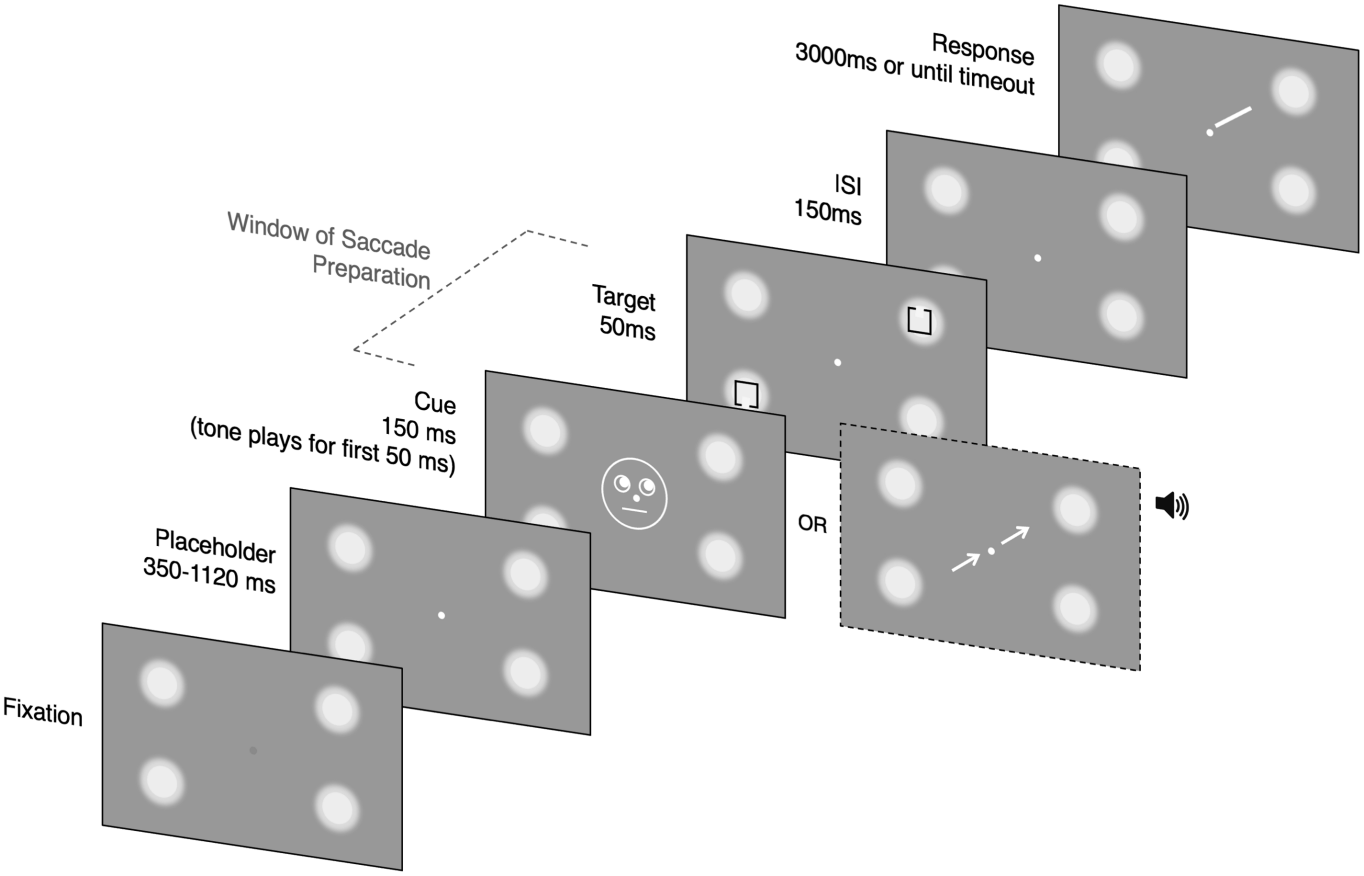
Trial Structure of a valid trial for experiment 1a (gaze-cue) and experiment 1b (arrow cue). Note that the trial progresses from fixation screen in lower left-hand corner to response screen in upper right-hand corner. Note the tone onset was simultaneous with cue onset but played for 50 ms only. The cue was immediately proceeded by two Landolt squares presented along one diagonal for 50 ms. At the time of target onset, participants were not aware which of the two stimuli was the to-be discriminated square and which the distractor. This was determined by a response cue presented after target offset and an interstimulus interval of 150 ms. The grey dashed line indicates the time period in which saccade preparation is assumed to occur. In this time period, participants are preparing an eye movement toward the instructed location, but eye gaze remains at fixation.

Across both experiments, we used an adapted cueing paradigm employed by Parker and colleagues (2021; see Figure 2). The first frame of the trial displayed a green fixation circle and four luminance circle placeholders (4.19 degrees diameter) positioned 9.1 degrees from the center of the screen in all four corners of the display. On dual task trials, participants were required to maintain gaze on the fixation circle while the space bar was simultaneously pressed to begin the trial. In the fixation condition, participants were instructed to maintain fixation while the space bar was pressed to commence the trial. The second frame displayed a white fixation circle and the placeholders for a variable duration. The duration of this frame was drawn randomly from an exponential distribution with a minimum of 350 ms. If the selected duration exceeded 1120 ms, then the trial would terminate and participants would receive the feedback “TIME OUT.” This type of catch trial formed 10% of all trials and were designed to ensure participants had a flat hazard rate with respect to the beginning of the trial (Ghose & Maunsell, 2002).

On trials in which the maximum duration was not exceeded, a tone was played for 50 ms. This tone served as the saccadic go-signal on dual task trials, indicating to participants to begin moving their eyes toward the instructed location. At the same time as the tone onset, a cue was displayed in the center of the screen and remained onscreen for 150 ms. In experiment 1a, this cue was a schematic of a face. This was created by using one large circle (3.35 degrees in diameter), with two smaller circles for the eyes (0.7 degrees in diameter). Inside the smaller circles were two filled circles or pupils that could be directed toward one of four placeholder locations (0.2 degrees in diameter). The fixation circle served as the nose on the face, whereas a line (0.3 degrees in length) was positioned underneath to represent the mouth. In experiment 1b, spatial attention was directed covertly by two arrows (1.6 degrees in length) positioned 0.4 degrees either side of the fixation circle along a diagonal. Again, the arrows were directed toward one of the four placeholder locations.

This was followed immediately by the onset of the target and distractor, two Landolt squares (0.9 degrees × 0.9 degrees) with a small gap positioned in the top or bottom side of the square displayed for 50 ms. One stimulus was presented in the placeholder indicated by the cue and the other was presented directly opposite it. Following a 150 ms offset, a response cue, a diagonally oriented line (1.0 degrees in length) indicated which stimulus the participant must report the location of the gap.

Participants were required to indicate whether the gap was in the upper or lower edge of the square by pressing the “up” or “down” key, respectively. On 50% of the trials, both stimuli had the same gap location, whereas on the remaining 50% of trials the gap location was in the opposite side. Valid trials were those where the to-be discriminated target appeared at the location indicated by the preceding cue. An invalid trial was where the target appeared in the location diagonally opposite the preceding cue. Trials timed out if no response was made after 3000 ms. Participants were given the feedback “correct” or “incorrect” about their decision. Gap width was controlled by a QUEST staircase procedure, which adjusted the width to an 82% accuracy threshold (Watson & Pelli, 1983). Gap location was jittered randomly along the side of the square.

### Procedure and design

All tasks took place in a dark room on an LED monitor (with a refresh rate of 120 Hz) positioned 104 cm from the front edge of the desk. In the dual task, a SR Research Desktop Mount Eyelink 1000 eye tracker, sampling at 500 Hz, was positioned in front of the screen and monitored the gaze position of the participants right eye throughout the task. The participant's head was stabilized throughout the task by a chin and forehead rest positioned at the front edge of the desk. As the fixation task did not require any eye movements, head position was not stabilized.

Across both experiments there was a between subjects’ factor of task. In the dual task condition, participants were required to simultaneously move their eyes to an instructed location and complete the discrimination decision. Participants in the fixation task condition were only required to complete the discrimination decision and were instructed to maintain fixation throughout the task. Consequently, the fixation task had only one within subject factor of cue validity. The fixation task consisted of 32 practice trials, followed by 192 trials of staircase procedure and 320 experimental trials. During the staircase procedure two interleaved staircases adjusted the gap width of the Landolt square to an 82% threshold. The staircase procedure was included so that accuracy would not be at ceiling. This was necessary to ensure that we had a sufficiently high error rate to enable modelling (Heathcote et al., 2019). The outcome of this block set the gap width maintained throughout the main experiment.

The dual task followed a 2 × 2 factorial design. There were two levels of cue validity (valid versus invalid) and two levels of saccade congruency (congruent versus incongruent). Saccade congruent trials were those in which eye movements were directed in the same location as the discrimination target. Whereas saccade incongruent trials were those where eye movements were directed to the location opposite the discrimination target.

Saccade instructions for the dual task were blocked such that participants were told at the outset of the block whether to move their eyes toward the upper left, upper right, lower left, or lower right placeholder upon the onset of the tone. The order of saccade direction was varied using a lattice square design across every fourth participant. Consistent with previous designs, 50% of dual task trials eye movements were directed along diagonals orthogonal to the cue and target (Parker et al., 2021). Although these trials were not of interest in the present study and thus excluded from data analysis, they were included in the design of the experiment to ensure the participants did not inadvertently associate saccade instruction with the upcoming location of the target (Parker et al., 2020b). For example, we wanted to exclude the possibility that participants could learn that stimuli will always appear along the same diagonal as the saccade target. The dual task consisted of 128 practice trials, followed by 256 trials of a QUEST staircase procedure, as outlined above and 768 experimental trials (384 trials included in our final analysis).

### Gaze data analysis

During dual task trials, eye movements were monitored both online and offline. Online monitoring was used to ensure that the participants maintained fixation until the saccadic go-signal and to provide them with feedback about their eye movements. If the eyes moved more than 1.17 degrees from the fixation circle before the onset of the tone, the trial was terminated and repeated. Offline analysis of gaze-data was used to determine saccadic onset. In order to detect saccade onset, eye position data at each time point was smoothed with a Gaussian. The average of five neighboring time points was then used to compute saccade velocity. Saccade onset was detected when eye velocity exceeded the median velocity by more than five standard deviations (SDs) for at least 8 ms (Engbert & Kliegl, 2003).

The trial was correct with respect to eye movements, if the landing position of the saccade fell within the directed placeholder. Incorrect eye movement trials were eliminated from analysis. This led to the exclusion of 10.8% of trials in experiment 1a and 15.2% of trials in experiment 1b. Similarly, trials in which eye movements were initiated before target offset were also excluded. This was to ensure that only trials in which the eyes were at fixation while the target was onscreen were compared. In experiment 1a, this led to the exclusion of 4.5% of trials and 3.4% of trials in experiment 1b. Rates of exclusion were similar across conditions. As is typical within the saccadic dual task literature, any trial where saccades were initiated more than 450 ms after target onset were excluded (Born, Ansorge, & Kerzel, 2013; Castet et al., 2006; Moehler & Fiehler, 2014; Moehler & Fiehler, 2015; Moehler & Fiehler, 2018). This was done to ensure that saccade preparation occurred simultaneously with target onset, rather than sequentially. In experiment 1a, this led to 0.5% of trials excluded from the analysis and 0.8% of trials in experiment 1b.

## Results

### Analysis of observed variables

**Figure 3.**
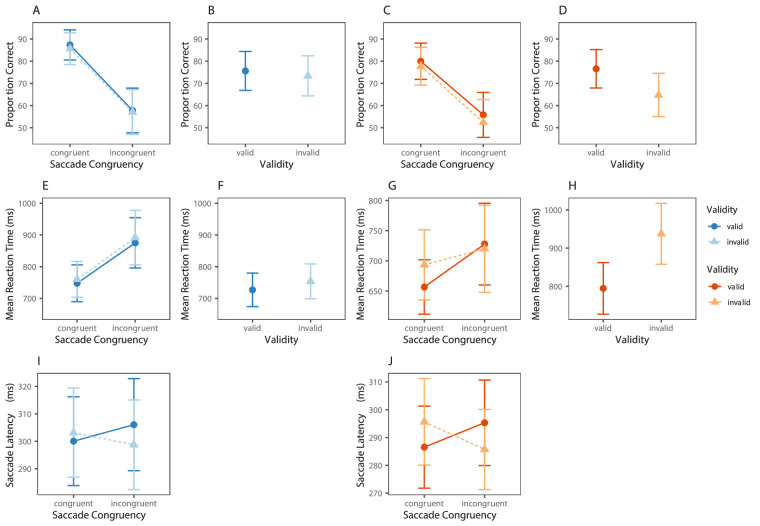
Data for experiments 1a and 1b. Note the blue graphs on the left represent results from experiment 1a (gaze cue). Orange graphs on the right denote results from experiment 1b (arrow cue). Top row represents accuracy results from (**A,** **C**) dual task and (**B,** **D**) fixation task. Second row represents reaction time results for the (**E,** **G**) dual task and (**F,** **H**) fixation task. Third row (**I,** **J**) represents saccade latency data from the dual task only. In all graphs points denote subject means, and error bars represent within subject standard errors of the mean.

Prior to the evidence accumulation modeling analysis, we first analyzed observed measures, which we define as variables directly measured in the task. This included a separate analysis of accuracy, RT, and saccade latency. These analyses were conducted using a Bayesian estimation approach to multilevel modeling (McElreath, 2020). As these analyses were not the main focus of this paper, we give a brief overview of these findings below. Raw data plots are shown in Figure 3 and full details of the analysis and results can be found in supplementary materials (see Supplementary Figures S1-S6 and Supplementary Table S2).

#### Experiment 1a

##### Fixation task

There was a cue validity effect in both accuracy and RTs in the expected direction. Responses were approximately 2% less accurate and 20 ms slower when the gaze cue was invalid relative to valid (see Figures 3B, 3F).

##### Dual task

On dual task trials, participants were, on average, approximately 30% less accurate (see Figure 3A) and 110 ms slower to make a button press response (see Figure 3E) when preparing an eye movement away from the upcoming location of the target relative to towards the target. The modeling procedure also revealed an effect of cue validity in both accuracy and RT measures in the expected direction (see Figures 3A, 3E). Note although the cueing effect is difficult to see in these figures, inspection of the posterior distribution for the full model in the supplementary materials revealed evidence of a cueing effect in both accuracy and RT of a similar magnitude and direction to a number of previous studies (Driver et al., 1999; Friesen & Kingstone, 1998; Friesen & Kingstone, 2002). When taken together, the results of the current study, combined with the finding that previous gaze-cueing studies show similar cueing effects, suggest that there is evidence of a cue validity effect in both accuracy and RT. Furthermore, there was little evidence of an interaction between saccade congruency and cue validity in either accuracy or RT.

In contrast, the modeling procedure on saccade latencies revealed evidence of an interaction between cue validity and saccade congruency. This interaction, which has been reported in previous studies, is best thought of as a cue-conflict effect in the motor execution of a saccade (Parker et al., 2020a; Parker et al., 2020b; Parker et al., 2021). That is, participants are slower to initiate eye movements to locations when they conflict with the direction of the cue (invalid-congruent/valid-incongruent) compared to when they do not (valid-congruent/invalid-incongruent; see Figure 3I). Please see supplementary materials (Supplementary Discussion section for Analysis of Observed Variables) for a full explanation of these findings.

#### Experiment 1b

##### Fixation task

As can be seen in Figures 3D and 3H, our results confirmed that there was a cue validity effect in experiment 1b in both accuracy and RT measures. Responses were approximately 12% less accurate and 200 ms slower when the target was preceded by an invalid relative to valid arrow cue.

##### Dual task

Analysis of accuracy measures revealed that responses were, on average, 23% less accurate on trials in which eye movements were being prepared away from the upcoming location of the target relative to toward. In addition, participants were approximately 3% less accurate on the Landolt gap discrimination task when the arrow cue was invalid relative to valid (see Figure 3C). Again, there was little evidence of an interaction between these two factors in accuracy measures specifically.

In contrast, analysis of RT revealed evidence to suggest that there was an interaction between saccade congruency and cue validity. As can be seen in Figure 3G there appeared to be an influence of saccade congruency on valid but not invalid trials (an alternative interpretation is that there is a cueing effect evident on congruent but not incongruent trials).

There was likewise evidence to suggest that there was an interaction between saccade congruency and cue validity in saccade latency measures. Again, participants were slower to initiate eye movements on trials in which the cue and eye movement direction conflicted relative to when they were aligned (see Figure 3J; see discussion of Analysis of Observed Variables in supplementary materials for a full explanation).

### Evidence accumulation modeling

#### Model specification

We fit the LBA model to each participants’ data for both the fixation and dual task trials for experiments 1a and 1b. LBA models have one accumulator for each response, each with potentially different parameter values. In this design, that meant that there was one accumulator for a target with a gap in the top edge and another for a target with a gap in the bottom edge (see Figure 1). Each accumulator possessed the following parameters; start point (*A*), representing the amount of evidence in each accumulator at the beginning of a decision; threshold (*b*) which represents the amount of evidence necessary to trigger a decision (in the present study reported in terms of the difference between the top of the start point distribution and the response threshold, *B* = *b* – *A* ≥ 0); drift rate (*v*) which represents the rate at which evidence for a response accumulates, capturing both quantity and quality of information accumulation; and non-decision time *T_er_* ≥ 0, the amount of time it takes for all other processes that fall outside the decision, such as stimulus encoding and motor responding.

Separate models were fit for each task for experiments 1a and 1b. Model specification was informed by prior literature (Parker et al., 2020a; Parker et al., 2020b; Parker et al., 2021) and outlined in our preregistration. Specifically, for the fixation task, thresholds were allowed to vary by response (up/down), whereas mean drift rate was allowed to vary by cue validity (valid/invalid) and the match between accumulator and stimulus. If the stimulus had a gap in the top edge, for example, then the accumulator for the “up” response would be the TRUE or matching accumulator, whereas the accumulator for the “down” response would be the FALSE or mismatching accumulator. In this way, the difference between the TRUE and FALSE accumulator captured both the quantity and quality of information about a decision (Lewandowsky & Oberauer, 2018). The larger the difference between accumulators the greater the quality of evidence accumulating in favor of that decision. Given these characteristics of drift rate, we took as our main dependent variable the difference between the true and false drift rate. The standard deviation for the true accumulator was allowed to vary by the match factor, whereas the standard deviation for the mismatching accumulator was held constant at one in order to make the model identifiable (Donkin et al., 2009). We estimated a single value for *A* and *T_er_*, as we had no reason to expect these values to differ by accumulator.

For dual task trials, thresholds were allowed to vary by both response and saccade instruction (top-left/top-right/bottom-left/bottom-right). Whereas mean drift rate was allowed to vary by both cue validity, saccade congruency (congruent/incongruent), and the match between accumulator and stimulus. All other aspects of the model specification were identical to the fixation task.

#### Model estimation

Model estimation was carried out in a Bayesian manner using the DMC software (Heathcote et al., 2019). Full details of priors and sampling methods are provided in supplementary materials and all the code required to run the analyses is available on the OSF (https://osf.io/fe9ds/). Priors were chosen to have little influence on estimation (graphical example of how posteriors were updated relative to priors in experiment 1a provided in Supplementary Figure S7). Sampling occurred in two steps. First, sampling was carried out separately for each individual participant for the fixed effects. The results of this step then provided the starting points for the full hierarchical model. Inspection of graphical summaries revealed the model to provide a good account of all major aspects of the data and cumulative distribution functions comparing experimental data with the model can be seen in Supplementary Figures S8 – S11.

#### Parameter estimates

To make inferences about the magnitude of the saccade congruency and cue validity effects across task conditions, we compared differences in parameter estimates across conditions. Specifically, for within subject effects, we calculated differences for each posterior sample for each individual subject parameter and then averaged across individuals to obtain the distribution of group average differences. This comparison captures correlations that are inherit in a within-subjects contrast. However, as these tests provide only inferences about fixed effects (that is about the group of subjects measured), for between subject comparisons, such as those across eye movement task and cue type, we applied the same procedure to hyperparameters. These hyperparameters represent the posterior distribution for population-level parameters in the hierarchical modeling procedure and can provide inferences about future subjects. As hyperparameters do not partial out between subject variation, credible intervals are wider for these comparisons compared to within-subject comparisons (Heathcote et al., 2019). To summarize the distribution of parameters of interest, we report the median of the posterior distribution together with 95% quantile interval of the posterior distribution in square brackets below.

#### Experiment 1a

##### Thresholds

In both the fixation and dual task versions of experiment 1a, there was little evidence that the threshold parameter varied by response (up or down), we therefore collapsed across this factor to estimate an overall threshold for the fixation task (0.90 [0.81, 0.96]).

In the dual task, we also collapsed across the eye movement instruction variable (which corner of the display participants were required to direct an eye movement toward) to provide an overall estimate of threshold (0.94 [0.91, 0.97]; see the Table 1 and the Comparison Across Task section for a comparison of these thresholds in hyperparameters).

**Table 1.** Median values and 95% credible intervals of the posterior distribution of parameter estimates in experiments 1a and 1b.

	Within subjects’ parameters			
	Gaze		Arrow	
	Fixation	Dual	Fixation	Dual
Cue	0.12 [0.05, 0.20]	0.10 [0.01, 0.20]	0.60 [0.50, 0.71]	0.14 [0.05, 0.23]
Saccade	NA	1.59 [1.49, 1.69]	NA	1.08 [0.98, 1.18]
B	0.90 [0.81, 0.96]	0.94 [0.91, 0.97]	1.47 [1.43, 1.52]	0.82 [0.79, 0.86]
	Hyper parameters			
	Gaze		Arrow	
	Fixation	Dual	Fixation	Dual

Cue	0.12 [−0.11, 0.37]	0.10 [−0.18, 0.39]	0.60 [0.25, 0.95]	0.14 [−0.14, 0.42]
Saccade	NA	1.58 [1.30, 1.87]	NA	1.08 [0.79, 1.35]
B	0.89 [0.75, 1.00]	0.94 [0.90, 0.98]	1.45 [1.25, 1.62]	0.79 [0.69, 0.86]

*Note* *:* The table provides median values for the posterior distribution of the parameter estimates of interest, with 95% credible interval displayed in square brackets. The top part of the table calculates these values within-subjects and therefore accounts for correlations inherent to this type of comparison. The bottom part of the table computes the values on hyperparameters for between-subject comparisons and population-level inferences. Cue = differences between valid and invalid in drift rate difference (true – false drift rate). Saccade = difference between congruent and incongruent conditions in drift rate difference. B = threshold estimate. NA, not applicable.

##### Drift rate

In order to quantify the relative contribution of both saccade congruency and cue validity within and across tasks, we computed the difference between the TRUE and FALSE drift rate (drift rate difference) as our main dependent measure. The cueing effect was computed by subtracting the drift rate difference for valid trials from invalid trials. The saccade congruency effect was likewise calculated by subtracting the drift rate difference on congruent trials from incongruent trials.

*Fixation task*. Experiment 1a revealed an effect of cue validity in line with predictions. Inspection of the posterior distribution of the cue validity effect in drift rate difference revealed a mostly positive distribution with values largely above zero (0.12 [0.05, 0.20]). The quality of evidence accumulating from the target was higher when preceded by a valid gaze cue (1.15 [1.09, 1.25]) relative to an invalid gaze cue (1.03 [0.97, 1.11]).

**Figure 4.**
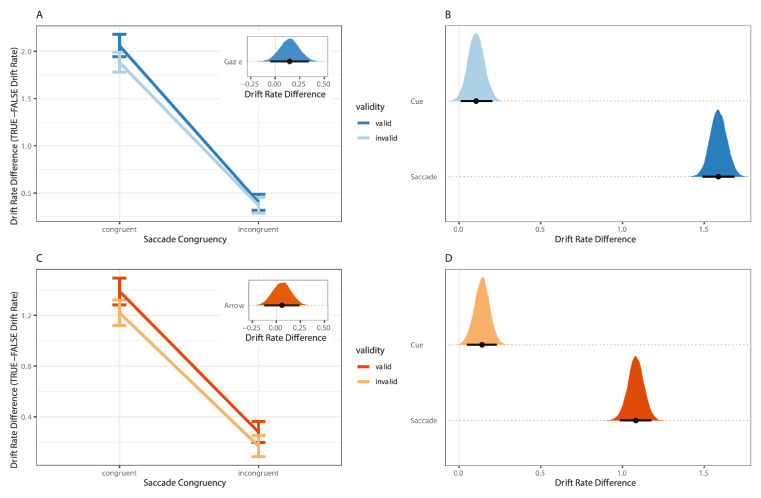
Within subject parameter estimates for experiments 1a and 1b dual-task conditions. Note the graphs on the left-hand side of the figure represent median values of the posterior distribution for the saccade congruency and cue validity effect in (**A**) experiment 1a and (**C**) experiment 1b. Error bars representing 95% credible intervals. Insets show the posterior distribution of the difference in the cueing effect between each level of saccade congruency. That is, we subtracted the magnitude of the cueing effect (valid – invalid) on congruent trials from incongruent trials. Graphs on the right-hand side of the figure plot the magnitude of the cue validity (valid – invalid) and saccade congruency (congruent – incongruent) effect in drift rate difference for experiment 1a (**B**) and experiment 1b (**D**) dual task. Note that the points are median values, whereas the black lines represent 95% credible intervals.

*Dual task*. Inspection of the posterior distributions revealed an influence of saccade congruency on dual task trials (1.58 [1.48, 1.68]). The distribution of which was entirely positive and above zero (see Figure 4B). The quality of evidence accumulation was higher on trials in which eye movements were prepared toward the target (1.97 [1.89, 2.05]) relative to away from the target (0.39 [0.33, 0.45]).

There was also a mostly positive effect of cue validity, with a large portion of the posterior distribution falling above zero (0.10 [0.01, 0.20]; see Figure 4B). The quality of information accruing from the target was greater on trials where the target was preceded by a valid gaze cue (1.23 [1.16, 1.31]) relative to invalid gaze cue (1.13 [1.06, 1.19]).

To assess whether there was any evidence to suggest that the saccade congruency and cue validity effects interacted, we computed the size of the cue validity effect (valid or invalid) at each level of saccade congruency and compared the relative magnitude of these effects (see Figure 4A). We then subtracted the magnitude of the cueing effect on congruent trials from incongruent trials and inspected the resulting posterior distribution of this term. As can be seen in Figure 4A, the posterior distribution of this difference of differences shows clear overlap with zero. Although the point estimate was above zero, the 95% quantile interval showed credible values falling both above and below zero. Given the posterior distribution considers the degree of uncertainty around the interaction term, we take this to suggest that there was little evidence of an interaction between saccade congruency and cue validity on dual-task trials. Additionally, given that this was a test of a novel interaction effect, rather than a replication of prior similar effects, we think it is most appropriate to interpret the result as lacking clear evidence of an interaction.

#### Experiment 1b

##### Thresholds

In the fixation task, we allowed thresholds to vary by response (up or down). Inspection of the posterior distributions revealed little evidence to suggest that thresholds varied by response, so we therefore collapsed across these measures to compute an overall threshold in the fixation task (1.47 [1.43, 1.52]).

Similarly, in the dual task version of experiment 1b, we collapsed across both response and eye movement instruction to calculate an average estimate of overall threshold (0.82 [0.79, 0.86]).

##### Drift rates

*Fixation task*. To quantify the magnitude of the cue validity effect in experiment 1b, we again calculated the difference in posterior estimates for valid compared to invalid trials. Inspection of the posterior distribution for the cue validity effect revealed a positive cueing effect, the values of which were wholly above zero (0.60 [0.50, 0.71]). The quality of evidence accumulation was higher on trials in which the target was preceded by a valid arrow cue (1.27 [1.19, 1.35]) relative to an invalid arrow cue (0.66 [0.50, 0.71]).

*Dual task*. There was a saccade congruency effect in drift rate difference in experiment 1b (1.08 [0.98, 1.18]). The quality of information accruing from the target on trials in which participants were preparing an eye movement toward the upcoming location of the target was greater (1.31 [1.23, 1.38]) than that evidence on trials in which an eye movement was prepared away from the upcoming location of the target (0.22 [0.17, 0.29]; see Figures 4C, 4D).

Inspection of the posterior distribution of the cueing effect similarly revealed an effect of cue validity, the values of which were largely positive and fell above zero (0.14 [0.05, 0.23]; see Figure 4D). That is, the quality of information accumulation was greater when the target was preceded by valid arrow cue (0.86 [0.77, 0.90]) relative to an invalid arrow cue (0.70 [0.63, 0.76]).

Again, in order to assess whether there was any evidence of an interaction between cue validity and saccade congruency in drift rate difference, we computed the magnitude of the cueing effect at each level of saccade congruency. Values in the posterior distribution were revealed to be close to zero with values falling both above and below zero (see inset of Figure 4C). There was little evidence of an interaction between saccade congruency and cue validity in drift rate difference for experiment 1b.

#### Comparison across eye movement task

We then sought to compare the magnitude of the cueing effect in both experiments 1a and 1b as a function of eye movement fixation and preparation task. To do so, we compared differences in the posterior distributions of effects of interest in hyper parameters across the fixation and dual task of each experiment.

#### Experiment 1a

**Figure 5.**
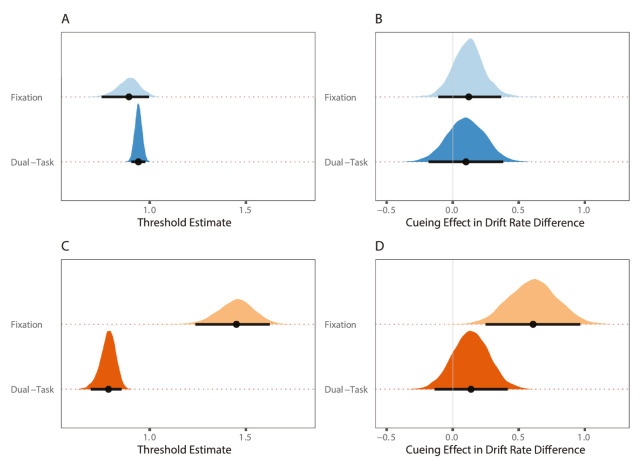
Between subject comparisons for experiments 1a and 1b (fixation versus dual task). Note the posterior distribution of the threshold estimates for the fixation and dual task in (**A**) experiment 1a and (**C**) experiment 1b. Posterior distribution of the cueing effect (valid – invalid) in drift rate difference for the fixation and dual task versions of (**B**) experiment 1a and (**D**) experiment 1b. Note across all graphs points are median values and thick black lines represent 95% credible intervals.

We first assessed how thresholds varied across tasks. Experiment 1a revealed there to be substantial overlap in the threshold distributions for the fixation and dual task (fixation: 0.89 [0.75, 1.00]; dual task: 0.94 [0.90, 0.98]; see Figure 5A).

Once differences in threshold were accounted for, we then assessed how the magnitude of the cueing effect varied as a function of task. This was achieved by first computing the difference between the true and false accumulator (drift rate difference) for the valid and invalid conditions, and then taking the difference between these conditions. As can be seen in Figure 5B, there was substantial overlap in the posterior distributions of the cueing effect between the fixation and dual task. The magnitude of the cueing effect was of a similar size regardless of whether participants were required to maintain fixation (without eye tracking) (0.12 [−0.11, 0.37]) or simultaneously prepare and execute eye movement (with eye tracking; 0.10 [−0.18, 0.39]).

#### Experiment 1b

Inspection of threshold estimates in experiment 1b revealed there to be a difference in the amount of evidence required to trigger a decision between tasks (see Figure 5C). Unexpectedly, participants required a higher degree of evidence to trigger a decision on the fixation task (1.45 [1.25, 1.62]) relative to the dual task (0.79 [0.69, 0.86]).

Inspection of the distribution of estimates for the cueing effect also revealed a difference in the magnitude of the cueing effect on fixation compared to dual task trials in experiment 1b. The median cueing effect was approximately four times larger in the fixation task (0.60 [0.25, 0.95]) relative to the dual task (0.14 [−0.14, 0.42]), but the 95% credible intervals still remained partly overlapping (see Figure 5D).

Given that these effects were not part of our preregistered hypotheses and therefore outside of the main focus of the paper, we cannot draw firm conclusions about them. Instead, we speculate on what might be driving these findings in the supplementary materials to help guide future research (see explanation in Discussion of Evidence Accumulation Modeling Analysis section of Supplementary Materials).

#### Comparison across experiments

As a final step, we sought to compare the magnitude of the saccade congruency and cue validity effect as a function of cue type (gaze versus arrow) by comparing the posterior distributions of the hyperparameters for experiments 1a and 1b. In order to do this, we computed the saccade congruency effect (congruent – incongruent) and cue validity effect (valid – invalid) in our measure of drift rate difference for both experiments 1a and 1b.

**Figure 6.**
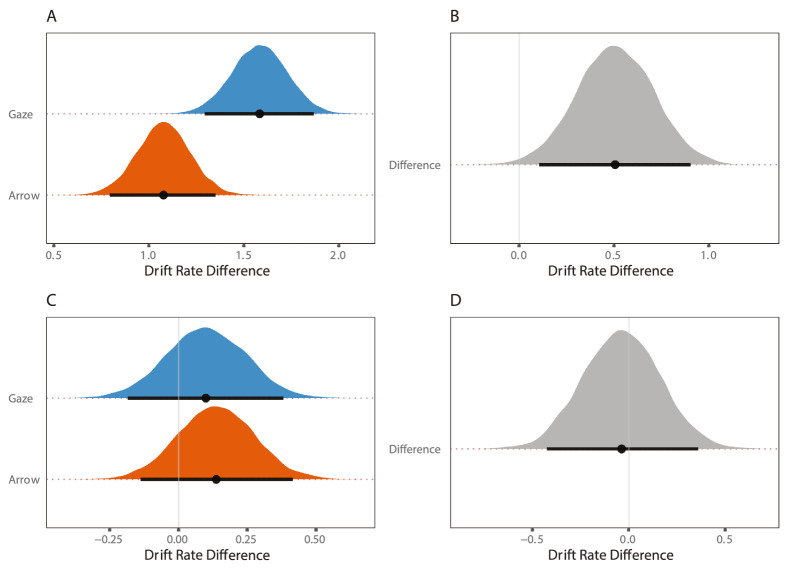
Magnitude of saccade congruence and cue validity effects for experiments 1a and 1b. Note the magnitude of (**A**) saccade congruency and (**C**) cue validity effects in experiment 1a (blue graph) and experiment 1b (orange graph). Difference between the posterior distributions for the (**B**) saccade congruency and (**D**) cue validity effect across experiments. Note points represent median values and black lines are 95% credible intervals from the posterior distribution.

As can be seen in Figure 6A, the median influence of saccade congruency on performance in the gaze cueing task (experiment 1a; 1.58 [1.30, 1.87]) was larger than the arrow cueing task (experiment 1b; 1.08 [0.79, 1.35]), whereas some overlap remained between the 95% credible intervals. The influence on preparing an eye movement toward versus away from the target on the quality of information accumulation was approximately 1.5 times greater in the gaze cueing relative to arrow cueing task.

In contrast, the distribution of posterior values for the cueing effect in experiments 1a and 1b substantially overlapped. There was a small influence of cue validity across both cueing tasks of a similar magnitude (experiment 1a; 0.10 [−0.18, 0.39] versus experiment 1b; 0.14 [−0.14, 0.42]; see Figures 6C, 6D).

## Discussion

The present study used evidence accumulation modeling to explore the relationship between covertly oriented spatial attention and eye movement preparation in social and non-social cueing tasks. The results supported our preregistered hypotheses and provide some of the first evidence to suggest that covertly oriented spatial attention and oculomotor preparation during gaze-cueing are dissociable and likely mediated by independent underlying mechanisms. Furthermore, the results also showed that the relationship between each type of orienting is similar in the context of both social (eye-gaze) and non-social (arrow) cues. By exploring the relationship between covert spatial attention and eye movement preparation during social and non-social cueing, this study sheds new light on how these types of orienting may operate within our social world. Specifically, these results establish that covert and overt orienting can make separable contributions to perception during social cueing and that the relationship between these types of orienting is similar for both social and non-social cueing.

### An evaluation of our preregistered hypotheses

In support of our first hypothesis, the results revealed a quantitatively distinct and separable contribution of saccadic and cue-based orienting during the gaze-cueing dual task. The quality of evidence accumulation (drift rate difference) was greater when targets were presented at the goal of a congruent compared to incongruent saccade and preceded by a valid compared to an invalid gaze-cue. That is, even on trials in which eye movements were prepared to the location diagonally opposite the target, the validity of the gaze cue still modulated performance. Although this finding is consistent with a growing number of studies that find (non-social) spatial attention can covertly shift away from the goal of an upcoming eye movement, usually during the early stages of saccade planning (Born et al., 2013; Castet et al., 2006; Moehler & Fiehler, 2014; Moehler & Fiehler, 2015; Montagnini & Castet, 2007; Parker et al., 2020a; Parker et al., 2020b; Parker et al., 2021), these results are the first to establish this also to be true when spatial attention is oriented covertly with a socially relevant gaze-cue.

We know of only one previous study in which the relationship between covert spatial attention and saccade preparation has been explored in the context of gaze cues (Morgan et al., 2014). Morgan and colleagues (2014) used an eye abduction paradigm to restrict eye movements in the direction of one hemifield, covert spatial attention was then directed by a gaze, arrow, or peripheral cue. The authors found only the gaze cueing effect to remain intact in the restricted hemifield and therefore concluded that it did not depend upon eye movements. Our results extend these findings by demonstrating that gaze-cueing can still independently influence performance even on trials in which eye movements are simultaneously prepared to the opposite diagonal. That is, there was still a measurably distinct contribution of gaze-cueing to performance despite a much larger saccade congruency effect evident in the same task. Furthermore, there was little evidence in the drift rate parameter to suggest that these effects interacted. In other words, the gaze-cue had the same impact on drift rate at congruent and incongruent saccade conditions. When taken together, these results provide robust evidence to suggest that gaze-cueing and oculomotor preparation are not only dissociable but make quantitatively distinct and independent contributions to perception within the same task.

In support of our second hypothesis, the magnitude of the gaze-cueing effect was similar across both eye movement conditions. Regardless of whether participants were instructed to maintain fixation or prepare and execute an eye movement, the gaze-cue made a similarly sized contribution to perception. Importantly, unlike prior work that did not use evidence accumulation modeling (Morgan et al., 2014), we were able to draw this conclusion after first accounting for possible differences in response caution (the threshold parameter). By doing so, the drift rate parameter in these studies provides a more unambiguous measure of attentional orienting following gaze-cues than has been previously possible (Driver et al., 1999; Friesen & Kingstone, 1998; Friesen & Kingstone, 2002; Ristic, Wright, & Kingstone, 2007). That is, whereas there were several differences between the dual and fixation tasks, such as the number of trials and the cognitive effort required for a dual-task, application of evidence accumulation modelling allowed us to account for the impact that these differences may have on performance and separate them from our measure of orienting. These results suggest that the influence of gaze-cueing is not obligatorily coupled to eye movements and that the mechanisms underlying covert and overt orienting to social stimuli can operate independently.

In support of our third hypothesis, our results revealed a similar relationship between eye movement preparation and covertly oriented spatial attention across both gaze- and arrow-cueing tasks. Specifically, regardless of cue type (eye-gaze versus arrow), there was a dissociable contribution of both saccade preparation and cue validity to task performance but little evidence for an interaction. Furthermore, the magnitude of the cueing effect across dual task trials was similar regardless of cue type. Such results are markedly similar to that previously reported for sudden onset peripheral cues (Parker et al., 2020a; Parker et al., 2021) and predictive centrally presented arrows (Parker et al., 2020b). It should be noted, however, that although the overall relationship between covert and overt attention appeared similar regardless of the social content of the cue, there were some differences between the two tasks that fell outside the scope of our hypotheses. On dual-task trials, there was a larger influence of saccade preparation in the gaze cueing task relative to the arrow cueing task, despite the saccade instructions being identical across both experiments. Similarly, the magnitude of the arrow cueing effect on fixation task trials was much larger than that evident on dual-task trials. Despite these differences, there was a similar pattern of dissociation between covert spatial attention and saccade preparation for both cue types. We take this as evidence consistent with the suggestion that the relationships between covert and overt attention are similar during social and non-social cueing.

It is noteworthy that Morgan and colleagues (2014) made a contrary conclusion by finding evidence to suggest that in contrast to arrow and peripheral cueing, gaze cueing was uniquely independent of eye movements. Morgan and colleagues’ (2014) conclusions, however, were drawn by comparing the magnitude of the cueing effect in mean RTs across blocked cue conditions. As a result, it is not possible to determine whether differences across cue type were due to the operation of covert spatial attention alone or confounded by differences in response caution that can occur across blocked conditions. Indeed, the results of the present study revealed response caution to vary as a function of cue type. In the current study, by using a computational modelling approach to study this question for the first time, we demonstrate that once these differences in response caution are accounted for, there is a similar dissociation between covert spatial attention and saccade preparation during both social and non-social cueing tasks.

### Implications for understanding the mechanisms of orienting to social cues

These findings have implications for those interested in how humans orient in response to social and non-social cues (Laidlaw, Foulsham, Kuhn, & Kingstone, 2011; Laidlaw et al., 2016), and also to vision researchers who have postulated that humans have developed these two distinct, yet complementary forms of orienting to subserve distinct social purposes (Hunt, Reuther, Hilchey, & Klein, 2019b; Klein, 2020). Vision researchers suggest that whereas eye movements have developed to communicate the locus of attention to others, covert shifts in attention facilitate perception and social interactions in situations where concealing eye movements is adaptive. For example, when individuals encounter an aggressor, it is adaptive to covertly monitor the actions of that person without revealing that they are the locus of our attention. Importantly, whereas vision researchers tend to make this claim in response to evidence of a dissociation between covertly oriented spatial attention and saccade preparation using distinctly non-social stimuli and paradigms, the results of the present study are consistent with such a conclusion in the context of orienting toward socially relevant gaze cues. That is, there was a separate and dissociable contribution of both covert spatial attention and eye movement preparation even when attention was directed by a gaze cue. Furthermore, the results revealed this pattern of dissociation to be invariant to the social content of the cue.

By combining evidence accumulation modeling with a saccadic dual task, the results of the present study allow us to draw inferences about orienting toward social cues that would otherwise be unavailable in a traditional separate analysis of accuracy and RT (Donkin et al., 2009; Donkin et al., 2011; Heathcote et al., 2019). In accounting for differences in response caution across blocked tasks, the computational approach has allowed us to quantify orienting towards social and non-social stimuli for the first time. This type of blocked task comparison, however, is not unique to research on social attention with many studies of social cognition utilizing similar designs. Given this, we believe the evidence accumulation approach has the potential to improve our understanding of the mechanisms that underlie social information processing more broadly.

### Limitations and constraints on generality

Although we endeavored to ensure participants were preparing an eye movement at the time of target onset during dual task trials, it is possible that participants may have delayed preparing an eye movement until after target onset. Whereas typical eye movements have a saccade latency of approximately 200 to 300 ms, participants in the current study had 600 ms from tone onset to execute the correct eye movement. It is therefore possible, on a small subset of trials (as indicated by a visual inspection of saccadic latency distribution, where only a small number of trials were in the tail end of the distribution), that participants may have delayed the preparation of an eye movement until the target was off the screen. We take the robust saccade congruency effect, however, as evidence against this proposition. Specifically, if participants were delaying an eye movement until after target offset, we would not expect the direction an eye movement was prepared toward to have any influence on performance. In contrast, we find across both experiments that the quality of evidence accumulation was greater on trials in which participants were preparing an eye movement towards, relative to away from the target.

Finally, it is important to acknowledge constraints on the generality of our findings (Simons, Shoda, & Lindsay, 2017). We interpret the results of the present study as evidence that the mechanisms that underlie covert and overt orienting are independent and largely invariant of the social content of the cue. However, we cannot rule out the possibility that this relationship may differ in other social contexts. In the present study, for example, we used a schematic gaze cue. Although this type of social cue is typical of gaze-cueing studies generally (Friesen & Kingstone, 1998; Friesen & Kingstone, 2002; Morgan et al., 2014), it bears little resemblance to the types of stimuli and circumstances which humans encounter in the real world (Hayward, Voorhies, Morris, Capozzi, & Ristic, 2017; Kingstone, 2009). Some authors have postulated that gaze cueing stimuli fail to capture many of the critical elements that make real eyes distinguishable from other stimuli such as arrows, with both cueing stimuli sharing similar features, such as communicating directionality (Birmingham & Kingstone, 2009). It is possible that the relationship between each type of orienting may differ in more ecologically valid experimental paradigms (Hayward et al., 2017). Indeed, we believe that future research would benefit from using a computational approach to explore the circumstances and degree to which orienting is modulated by these types of manipulations. The evidence accumulation modeling approach, outlined in the present study, provides a novel way to compare across manipulations in sociality.

## Supplementary Material

Supplement 1
