# Supplementary material for: Exploring the relationship between oculomotor preparation and gaze-cued covert shifts in attention
Source: J Vis. 2023 Mar 30;23(3):18. doi: 10.1167/jov.23.3.18 (PMC10067775; doi:10.1167/jov.23.3.18)
Supplement: Supplement 1 [file jovi-23-3-18_s001.pdf]

Exploring the relationship between oculomotor preparation and gaze-cued covert shifts in attention.

Supplementary Materials

Analysis of Observed Variables

In addition to our evidence accumulation modelling analysis, we first analysed accuracy, reaction time (RT) and saccade latency (time between saccadic go-signal and target onset). Analyses was conducted using a Bayesian estimation approach to multilevel modelling (McElreath, 2020). Specifically, we used the Bayesian modelling package "brms" to run multilevel models in the R (Bürkner, 2017). Given that task and cue type were between subject factors we fit models separately for all four tasks.

Models were built incrementally towards the most complex model. This meant that all fixed and varying effects that the design would permit were included in the full model (Barr et al., 2013). Model 0 for all three dependent variables was an intercept only model so that we could compare all subsequent models that included effects of interest to a model without any predictors. For the fixation version of Experiment 1a and 1b the full model (Model 1) included an effect of cue validity (invalid vs. valid) as a fixed effect. In the dual-task version of Experiment 1a and 1b Model 1 included a fixed effect of saccade congruency (incongruent vs. congruent). Model 2 added an effect of cue validity (invalid vs. valid). Model 3, the most complex model, included an interaction between saccade congruency and cue validity. Shifted lognormal models were used to fit both reaction time and saccade latency data, while accuracy data was fit with a Bernoulli model. Deviation coding was used for both the cue validity and saccade congruency factor (-0.5 for invalid/incongruent; 0.5 for valid/congruent). This meant that the intercept could be interpreted as the grand mean and fixed effects

interpreted in a similar way to an ANOVA. Priors were set using a weakly informative approach (see Table 1 for all priors used for modelling) (Gelman, 2006). The formula for the full model (Model 3) used to fit reaction time and saccade latency data is specified below:

$$\begin{gathered} \text{RT} \sim 1 + \text{ei} * \text{validity} + \\ (1 + \text{ei} * \text{validity} \mid \text{SID}), \\ \text{ndt} \sim (1 \mid \text{SID}) \end{gathered}$$

Note: RT = reaction time (ms); ei = saccade congruency (congruent vs. incongruent); validity = cue validity (valid vs. invalid); SID = subject identifier; ndt = non-decision time.

We explored our hypothesis by examining the posterior distribution for estimates of the most complex models. for the most complex models. Parameter estimates for Experiment 1a and 1b are depicted in Supplementary Figure S1 for the fixation task and Figure S3 for the dual task. Point estimates for all tasks are also provided in Supplementary Table 2. We also conducted model comparison which can be seen in supplementary figure S5 and S6.

Table 1
Priors used for models

Dependent Variable	Prior	Class	dpar
Accuracy	Normal (0, 1)	Intercept	
	Normal (0, 0.5)	sd	ndt
	Normal (0, 0.5)	b	
	Lkj (2)	cor	
Reaction Time	Normal (6.68, 0.5)	Intercept	
	Normal (5.99, 0.5)	Intercept	ndt
	Normal (0, 0.1)	b	
	Normal (0, 0.1)	sd	
	Normal (0, 0.1)	sd	ndt
	Normal (0, 0.1)	sigma	
	Lkj (2)	cor	
Saccade Latency	Normal (5.70, 0.5)	Intercept	
	Normal (5.19, 0.5)	Intercept	ndt
	Normal (0, 0.1)	b	
	Normal (0, 0.1)	sd	
	Normal (0, 0.1)	sd	ndt
	Normal (0, 0.1)	sigma	
	Lkj (2)	cor	

Note: dpar = distributional parameter; sd = standard deviation; b = fixed effect; cor = correlation; ndt = nondecision time.

Experiment 1a. Gaze Cue

Fixation-task

Accuracy. Results demonstrated a cue validity effect, that is responses were less accurate when the gaze cue was invalid (*M* = 73.4, *SD* = 44.2) relative to valid (*M* = 75.6, *SD* = 43.0).

Parameter estimates from the full model for the fixation task indicated a negative effect of cue validity (invalid vs. valid). Responses on the Landolt gap detection task were less accurate when the gaze cue was invalid relative to valid. Supplementary Figure S1 demonstrates that responses were approximately 2% less accurate when the cue was invalid relative to valid.

Reaction time. Visual inspection of reaction times similarly revealed a cue validity effect, such that Landolt gap decisions were slower when the gaze cue was invalid (*M* = 754 ms, *SD* = 270 ms) relative to valid (*M* = 727 ms, *SD* = 258ms).

Parameter estimates from the full model revealed that there was a positive effect of cue validity (see supplementary figure S2). Reaction times were slower when the gaze cue was invalid relative to valid. As can be seen in S1 responses were approximately 20ms slower when the gaze cue was invalid.

Dual-Task

Accuracy. Inspection of accuracy results of dual-task trials for Experiment 1a revealed an influence of saccade congruency. Responses were more accurate when participants were preparing an eye movement towards the target (*M* = 89.1, *SD* = 31.2) relative to away (*M* = 58.5, *SD* = 49.3). Similarly, upon visual inspection there was also an effect of cue validity such that responses were more accurate when the gaze cue was valid (*M*

= 74.5, *SD* = 43.6) compared to invalid (*M* = 73.1, *SD* = 44.4). There appeared to be little evidence of an interaction between the two factors.

The posterior distributions of the fixed effects of the full model (model 3) revealed a negative effect of saccade congruency (incongruent vs. congruent) and a smaller negative effect of cue validity (invalid vs. valid), with values from the distribution falling largely negative of zero. The distribution of the estimates for the interaction effect had a positive peak, but with values falling either side of zero. These results support the interpretation that cue validity and saccade congruency make distinct contributions to task performance. The results revealed people to be approximately 30% less accurate when preparing an eye movement away from the target location relative to towards (see supplementary figure S4) and 2% less accurate when the gaze cue was invalid relative to valid.

Reaction Time. There was similarly an influence of saccade congruency on reaction times. Responses were faster when the prepared eye movement was congruent (*M* = 760 ms, *SD* = 256 ms) with the saccade target relative to incongruent (*M* = 902 ms, *SD* = 370 ms). There appeared to be an influence of cue validity, with responses faster when the gaze cue was valid (*M* = 809 ms, *SD* = 311 ms) relative to invalid (*M* = 824ms, *SD* = 318ms).

The posterior distributions of the fixed effects of the full model (model 3) revealed a negative effect of saccade congruency (incongruent vs. congruent) and a smaller, but largely negative effect of cue validity. The majority of the posterior distribution for this parameter estimate fell and peaked negative of zero. Inspection of the distribution for the interaction term revealed that although the peak of the distribution was positive, values fell either side of zero. These results support an interpretation that cue validity and saccade congruency made distinct contributions to performance. The results revealed that people were approximately 110 ms faster to response when making an eye movement towards the target relative to away.

Saccade Latency. Visual inspection of Experiment 1a saccade latencies revealed that there was likely an interaction between the cue validity and saccade congruency effects.

Inspection of the posterior distribution of the parameter estimates for the full model confirmed this. The parameter estimates of both saccade congruency and cue validity peaked at zero with values falling either side of zero. The distribution of the parameter estimate for the interaction term was largely negative. Eye movements were slower to initiate in the opposite direction to the gaze cue (incongruent valid/congruent invalid) compared to the same direction as the gaze cue (congruent valid/incongruent invalid).

Experiment 1b Arrow Cue

Fixation-task

Accuracy. Accuracy on the Landolt Gap discrimination task was greater when the arrow cue was valid (M = 76.6, SD = 42.4) relative to invalid (M = 64.8, SD = 47.8).

Parameter estimates for the full model (model 1) demonstrated a largely negative effect of cue validity (invalid vs. valid). That is, responses were less accurate when the cue was invalid relative to valid. Participants were approximately 12% more accurate when provided with a valid arrow cue, relative to invalid (see supplementary figure S2).

Reaction Time. Visual inspection revealed a cue validity effect in reaction times. Responses were faster when the target was preceded by a valid arrow (M = 795 ms, SD = 331 ms) cue relative to invalid (M = 937 ms, SD = 391 ms) (see Figure 4H).

The posterior distribution for this parameter estimate revealed a largely positive effect of cue validity. As can be seen in Supplementary Figure S2 responses were approximately 200ms faster when the cue was valid compared to invalid.

Dual-Task

Accuracy. Visual inspection of accuracy results for Experiment 1b revealed what appeared to be an effect of saccade congruency. That is, responses to the gap decision task were more accurate when saccade preparation was congruent (*M* = 78.8, *SD* = 40.9) to the target, relative to incongruent (*M* = 54.1, *SD* = 49.8). There similarly appeared to be an effect of cue validity, such that responses were more accurate when the cue was valid (*M* = 67.8, *SD* = 46.7) relative to invalid (*M* = 65.3, *SD* = 47.6).

Inspection of parameter estimates for the most complex model (model 3) demonstrated a large negative effect of saccade congruency (incongruent vs. congruent). Figure 6D demonstrates that people were approximately 23% more accurate when preparing an eye movement towards the target, relative to away. There was also a smaller, but largely negative effect of cue validity. People appeared to be approximately 3% more accurate when the cue was valid relative to invalid. Values for the interaction term were close to zero, with values falling both above and below zero. The best interpretation of this estimate is that there was little evidence for an interaction between cue validity and saccade congruency in accuracy measures.

Reaction Time. There appeared to be an effect of saccade congruency in reaction times for Experiment 1b. That is, reaction times appeared to be faster when the eye movement plan was congruent (*M* = 675 ms, *SD* = 256 ms) with the upcoming location of the target relative to incongruent (*M* = 724 ms, *SD* = 342 ms). There also appeared to be an advantage of the arrow cue being valid (*M* = 686 ms, *SD* = 275 ms) relative to invalid (*M* = 704 ms, *SD* = 314 ms), however, this appeared to interact with saccade congruency.

Parameter estimates revealed that there was a largely positive effect of saccade congruency, as well as a smaller but still largely positive effect of cue validity. However, values for the interaction term were largely negative. The results are consistent with the suggestion that there may be an interaction between saccade congruency and cue validity in

reaction times for Experiment 1b (see supplementary Figure S4). However, supplementary figure S4 makes it apparent that the interaction is difficult to interpret. That is, it appears that on invalid trials there is a negligible influence of saccade congruency.

Saccade Latency. Inspection of saccade latencies revealed an interaction between saccade congruency and cue validity for Experiment 1b.

Parameter estimates of the most complex model supported this interpretation. Values for both the saccade congruency and cue validity effect were close to zero with values falling both above and below zero. Values for the interaction term, however, were largely negative. This finding, consistent with saccade latency results for Experiment 1a, support the interpretation that there was an interaction between saccade congruency and cue validity in saccade latencies. That is, eye movements were slower to initiate in directions opposite the cue, compared with in the same direction as the cue.

Discussion of Observed Variable Analysis: Explanation of Saccade Latency Interaction

It should be noted that our analysis of saccade latencies revealed an interaction between saccade congruency and cue validity in saccade latencies specifically. We believe that this effect is best interpreted as a conflict between eye movement direction and cue validity. That is, eye movements are slower to initiate when made in directions opposite the spatial cue, compared to in the same direction. Previous studies utilising a similar design as that in the present paper, demonstrate a markedly similar patterns of results. These effects have been found, through computational modelling, to be best characterised as a bottleneck in the motor execution of an eye movement, rather than evidence of a perceptual yoking between orienting mechanisms (Parker et al., 2020a, 2020b, 2021). Given the similarities

between designs, it is most likely that the saccade latency results in the present study are similarly indicative of this type of motor bottleneck.

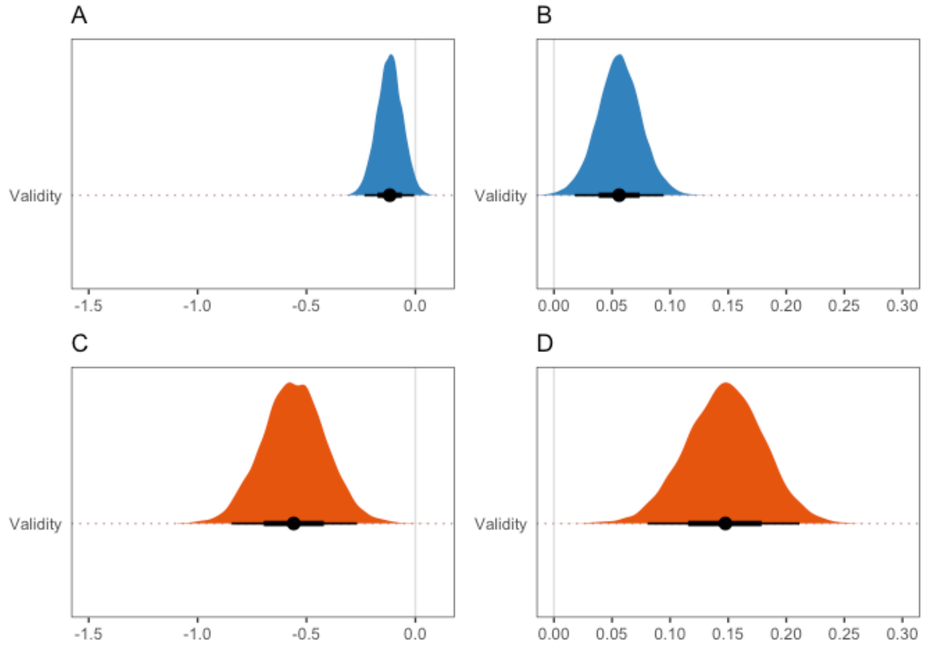

Supplementary Figure S1. Fixed effects for most complex model (Model 1) for (A/B) Experiment 1a and (C/D) Experiment 1b fixation-task. First column is for accuracy, second column is for RT. The x-axis for the first column is expressed in log odds, while the second column is expressed on the log(RT) scale. Note the point represents the median value of the posterior distribution for that estimate, the thicker line is the 66th percentile, while the thinner line is the 95th percentile of the distribution.

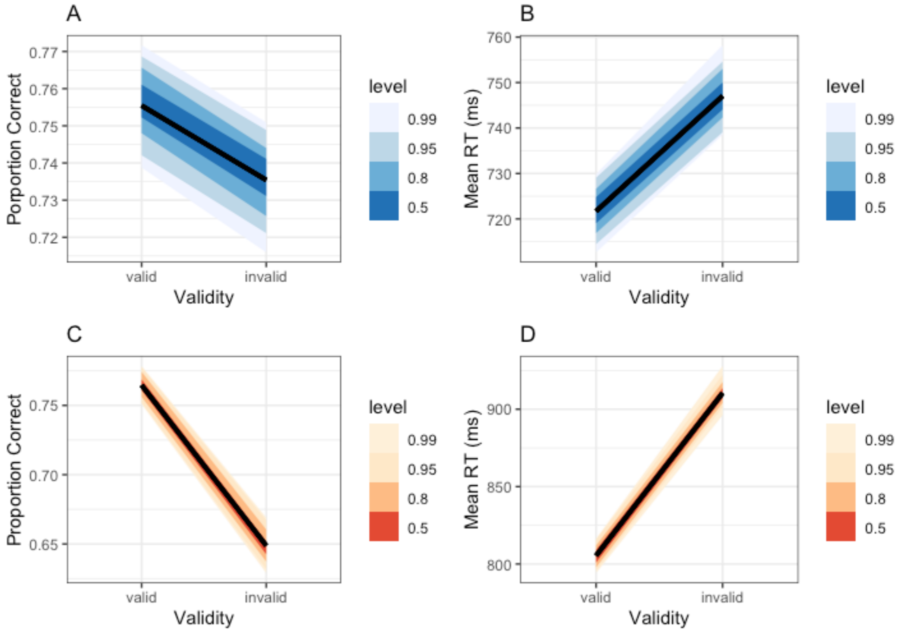

Supplementary Figure S2. Probability bands for 100 draws from the posterior distributions for the fixation task for (A/B) Experiment 1a and (C/D) Experiment 1b. First column represents accuracy, while the second column represents reaction time (ms).

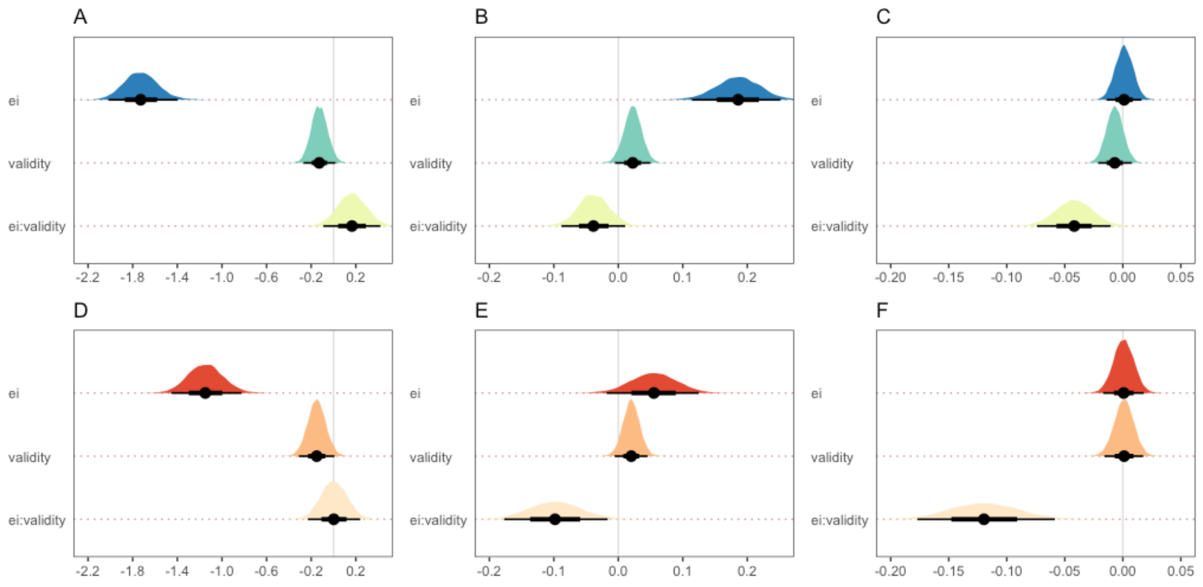

Supplementary Figure S3. Fixed effects of the most complex model (Model 3) for (A-C) Experiment 1a and (D-F) 1b. Graphs in the first column represents estimates for accuracy, second column are estimates for reaction times and final column is for saccade latency. The x-axis for the first column is expressed in log odds, while the x-axis for the second and third columns are expressed on the log(RT) scale. The saccade congruency parameter represents the difference between incongruent minus congruent, while the cue validity effect represents the difference between invalid minus valid. Note the point is the median value of the distribution, while the thicker line represents the 66th percentile and the thinner line is the 95th percentile of the distribution. ei = saccade congruency.

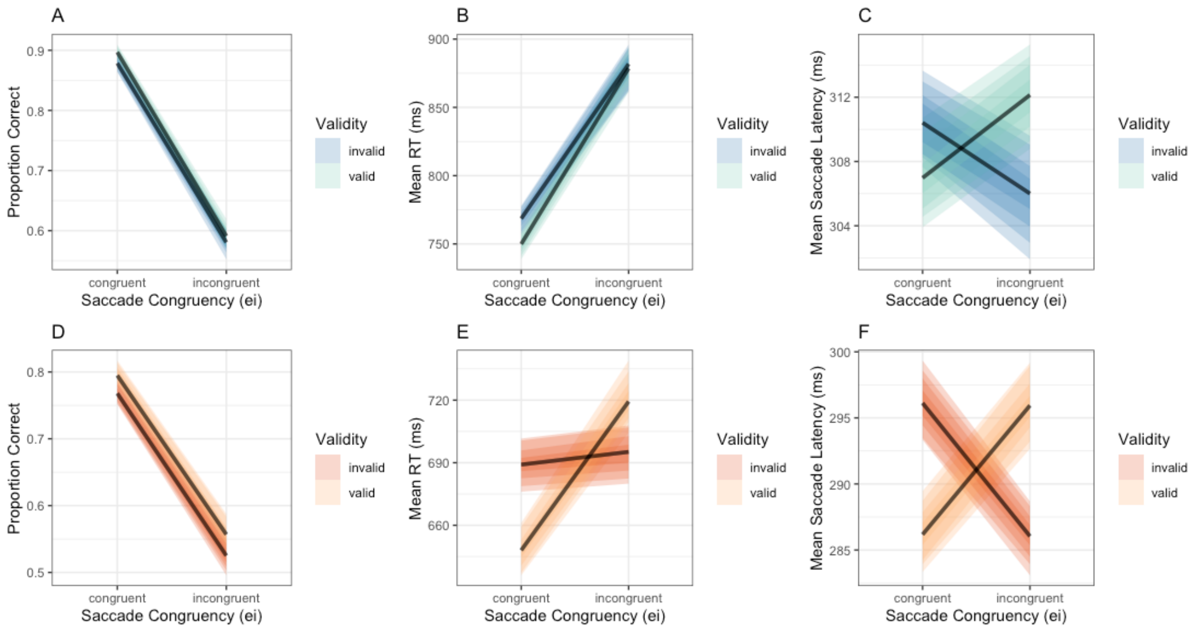

Supplementary Figure S4. Probability bands for 100 draws from the posterior distribution for the dual-task trials for (A-C) Experiment 1a and (D-F) Experiment 1b. The first column represents draws for accuracy, the middle column are probability draws for reaction times and the final column is the probability bands for saccade latency. (Supplementary)

Supplementary Table 2.
Posterior Median of Parameter Estimates for the Full Models in Experiment 1a and 1b.

Fixed Effect	Experiment 1a			Experiment 1b		
	Accuracy	RT	Saccade Latency	Accuracy	RT	Saccade Latency
			Fixation Task			
Intercept	1.10 [0.92, 1.30]	5.99 [5.88, 6.10]	NA[a]	0.95 [0.76, 1.12]	6.30 [6.17, 6.43]	NA
ndt Intercept[b]	NA	5.56 [5.39, 5.72]	NA	NA	5.32 [5.12, 5.51]	NA
Validity	-0.12 [-0.23, -0.00]	0.06 [0.02, 0.09]	NA	-0.56 [-0.84, -0.27]	0.15 [0.08, 0.21]	NA
			Dual Task			
Intercept	1.25 [1.05, 1.46]	6.04 [5.91, 6.17]	5.19 [5.05, 5.33]	0.76 [0.59, 0.92]	5.94 [5.81, 6.08]	4.94 [4.83, 5.07]
ndt Intercept	NA	5.73 [5.57, 5.88]	4.64 [4.47, 4.79]	NA	5.26 [5.01, 5.50]	4.89 [4.79, 4.99]
Validity	-0.13 [-0.27, 0.02]	0.02 [-0.01, 0.05]	-0.01 [-0.02, 0.01]	-0.15 [-0.31, 0.01]	0.02 [-0.01, 0.05]	0.00 [-0.02, 0.02]
Saccade Congruency	-1.73 [-2.02, -1.40]	0.19 [0.11, 0.25]	0.00 [-0.01, 0.02]	-1.15 [-1.45, -0.83]	0.06 [-0.02, 0.13]	0.00 [-0.02, 0.02]
Validity * Saccade Congruency[c]	0.17 [-0.09,0.42]	-0.04 [-0.08, 0.01]	-0.04 [-0.07, -0.01]	0.00 [-0.23, 0.23]	-0.10 [-0.18, -0.02]	-0.12 [-0.18, -0.06]

Note. Posterior medians are presented with 95% credible intervals. Model 1 was the full model for the fixation task, while Model 3 was the full model for the dual-task across both Experiment 1a and 1b.
[a] NA = not applicable (either because this variable does not exist for this task or model).
[b] ndt = non-decision time.
[c] Validity * Saccade Congruency = Interaction between the two effects.

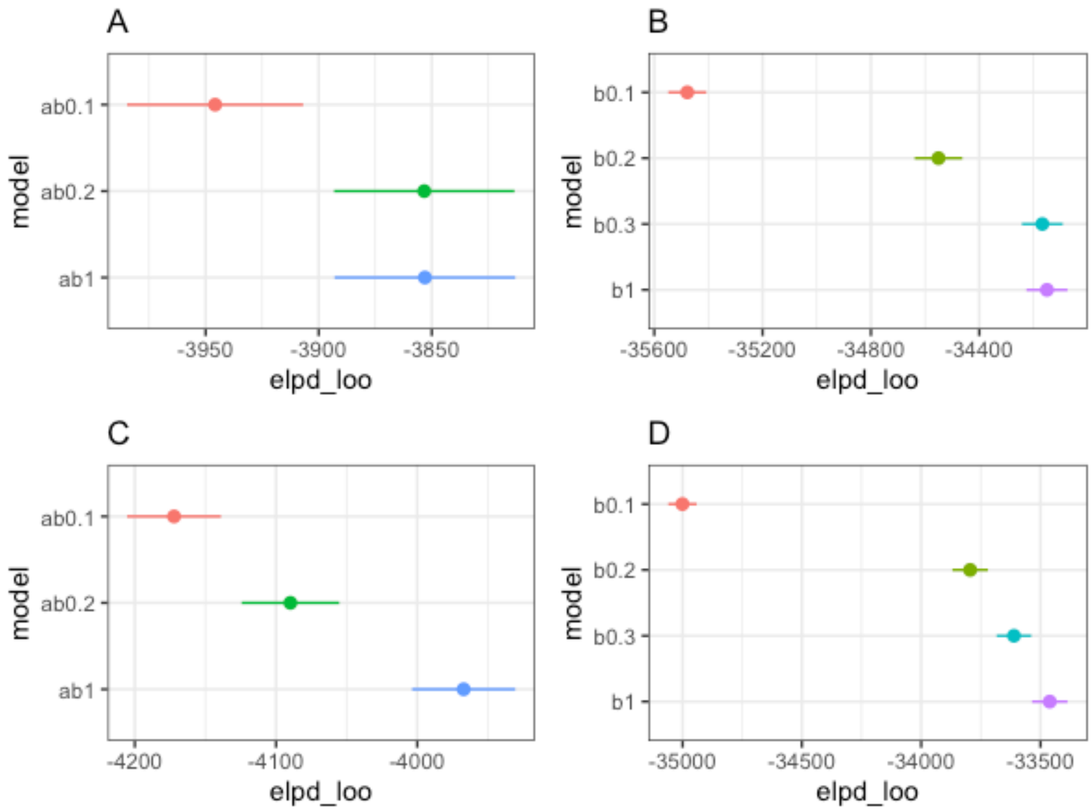

Supplementary Figure S5. Model Comparison for (A)-(B) Experiment 1a (gaze cue) and (C)-(D) Experiment 1b (arrow cue) fixation task. The first column is the model comparison results for accuracy models, and the second column is for reaction time models. Note: Model ab0.1 is the intercept only model, ab0.2 adds variation by participant. Ab1 (Model 1) is the full model and includes a fixed effect of cue validity. Model b0.1 is the intercept only model, b0.2 adds variation by participant, while b0.3 adds participant variation by the non-decision time parameter. b1 (Model 1) is the full model for the fixation task and includes a fixed effect of cue validity.

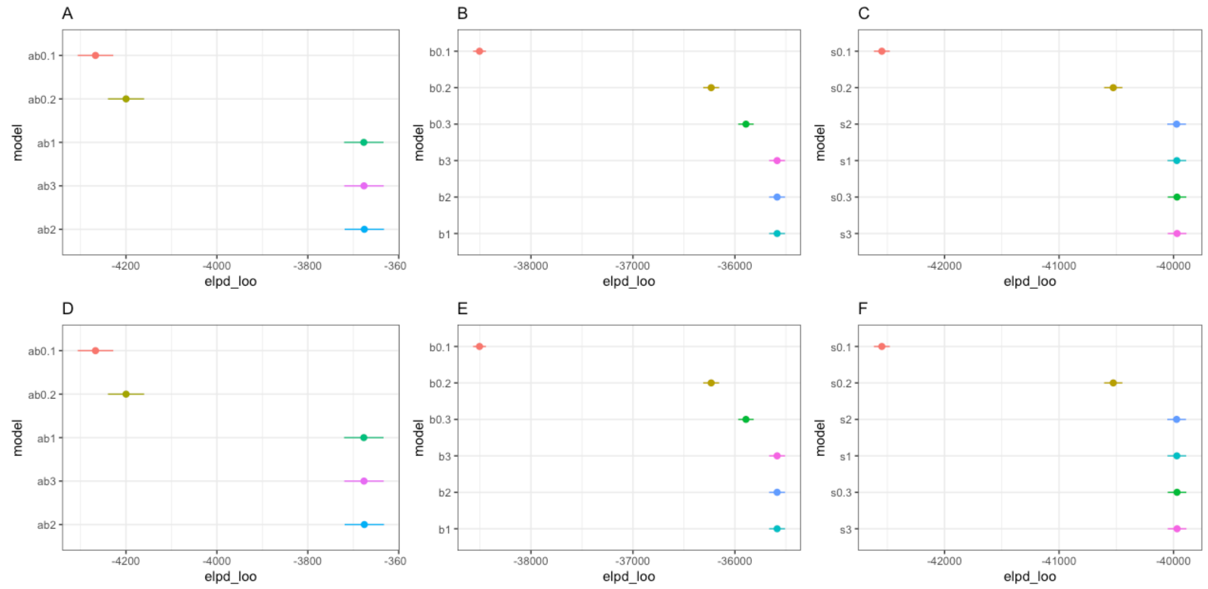

Supplementary Figure S6. Model Comparison for (A)-(C) Experiment 1a and (D) – (F) 1b for dual-task trials. The first column are the model comparison results for accuracy models (ab0 – ab3), the middle columns represent reaction time models (b0 – b3) and the final column is saccade latency models (s0 – s3)

Note: ab0.1 = Intercept only model; ab0.2 = adds variation by participant; ab1 = adds fixed effect of saccade congruency; ab2 = adds fixed effect of cue validity; ab3 = add fixed effect of interaction between saccade congruency and cue validity; b0.1 = Intercept only model; b0.2 = adds variation by participant; b0.3 = adds variation by participant in the non-decision time; b1 = adds fixed effect of saccade congruency; b2 = adds fixed effect of cue validity; b3 = adds interaction between cue validity and saccade congruency. Saccade latency model notations identical to reaction time (s0.1 – s3).

Linear Ballistic Accumulator Analysis

Sampling

For each model we used three times as many chains were used as model parameters. Sampling was carried out in two steps. First, sampling was carried out separately for individual participants in order to get reasonable start points for hierarchical sampling. The results of this step were then used as starting points for sampling the full hierarchical sample. During the initial burn-in-period there was a probability of .05 that a crossover step was replaced with a migration step. After burn in only crossover steps were used and sampling continued until the proportional scale reduction factor (R") was less than 1.1 for all parameters, and also the multivariate version was less than 1.1 (Brooks & Gelman, 1998). Hierarchical estimation assumed independent normal population distributions for each model parameter. Population-mean start points were calculated from the mean of the individual-subject posterior medians and population standard deviation from their standard deviations, with each chain getting a slightly different random perturbation of these values. Hierarchical sampling used probability .05 migration steps at both levels of the hierarchy during burn in and only crossover steps thereafter with thinning set at 10 (i.e., only every 10th sample was kept), with sampling continuing until R" for all parameters at all levels, and the multivariate R" values, were all less than 1.1. The final set of chains were also inspected visually to confirm convergence.

Priors.

Priors were chosen to have little influence on estimation. Priors were normal distributions that were truncated below zero for *B, A* and *sv* parameters, and truncated at 0.1s for the *t0* parameter (assuming that responses made in less than 0.1s are implausible). The *t0* parameter was truncated above by 1s. There were no other truncations, so the *v* prior was

unbounded. The prior mean for *B* was 1 and for *A* 0.5. The *v* parameter for the true accumulator was given a prior mean of 1, while the mismatching accumulator was given a prior mean of 0. The *sv* parameter for the matching accumulator had a prior mean of 0.5. The *t0* parameter had a prior mean of 0.3s. All priors had a standard deviation of 2. Mean parameters of population distributions were assumed to have priors of the same form as for individual estimation, and the standard deviations of hyper parameters were assumed to have exponential distributions with a scale parameter of one. Plots of prior and posterior distributions revealed strong updating (i.e., posteriors dominated priors), making it clear that the prior assumptions had little influence on posterior estimates (see S8 for an example of how the priors were updated for hyperparameters for Experiment 1a Fixation Task).

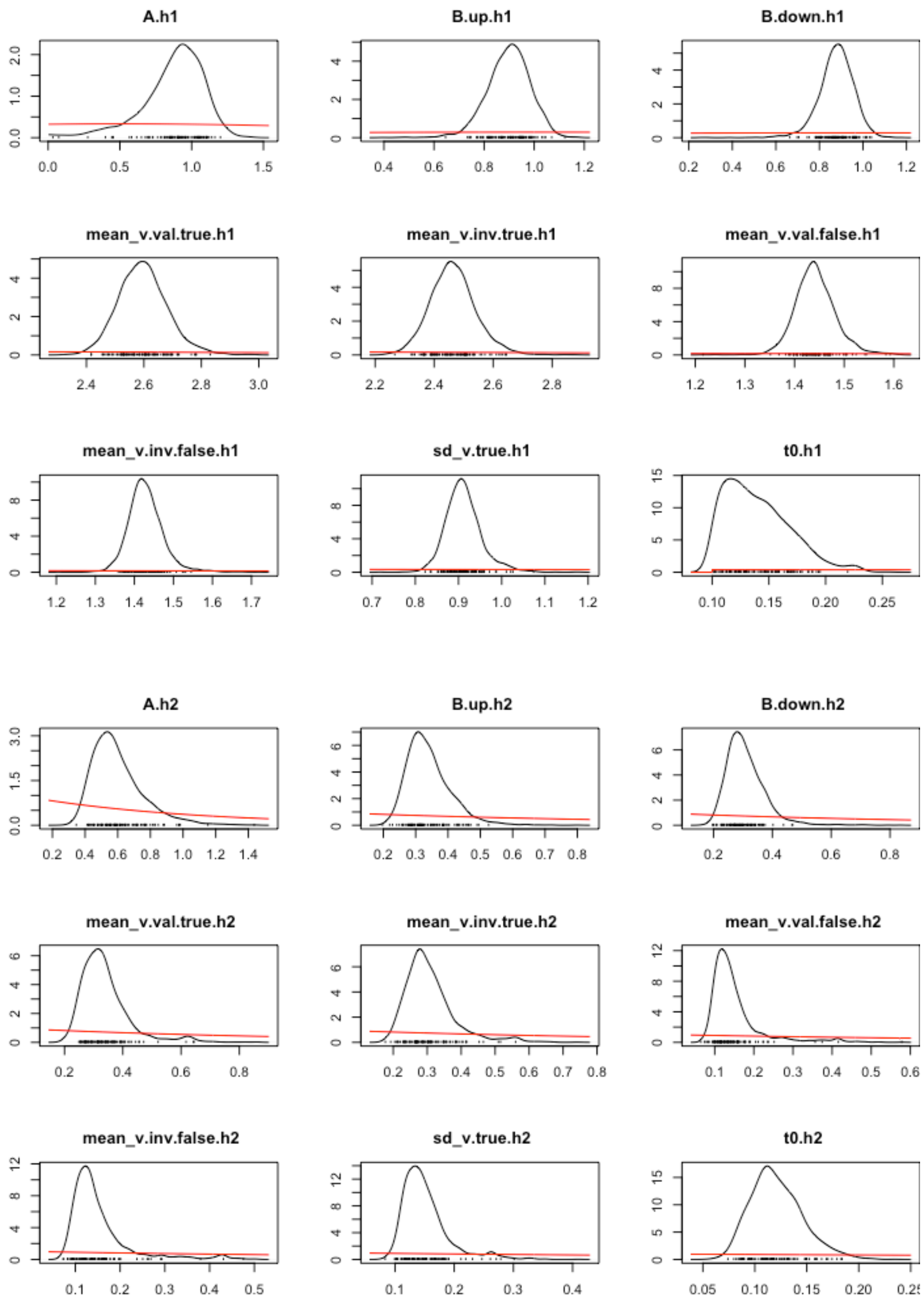

Supplementary Figure S7. Prior and Posterior Graphs for Experiment 1a Fixation Task. Red lines represent priors, while black lines represent posterior for Experiment 1a Fixation Task.

Model fit.

Supplementary Figures 8-11 display the fits of the LBA model to the data in terms of defective cumulative distribution functions (lines) and 10th, 30th, 50th, 70th and 90th percentiles (points from left to right) averaged over participants. Thick black lines and open points correspond to the data and the think grey lines solid black points to the model prediction averaged over posterior samples. The grey points correspond to percentile predictions for 100 randomly selected sets of posterior parameter samples, so their spread gives an idea of the uncertainty in the model's predictions. As can be seen from the figures the average fit of the selected LBA models for both Experiment 1a and 1b were good.

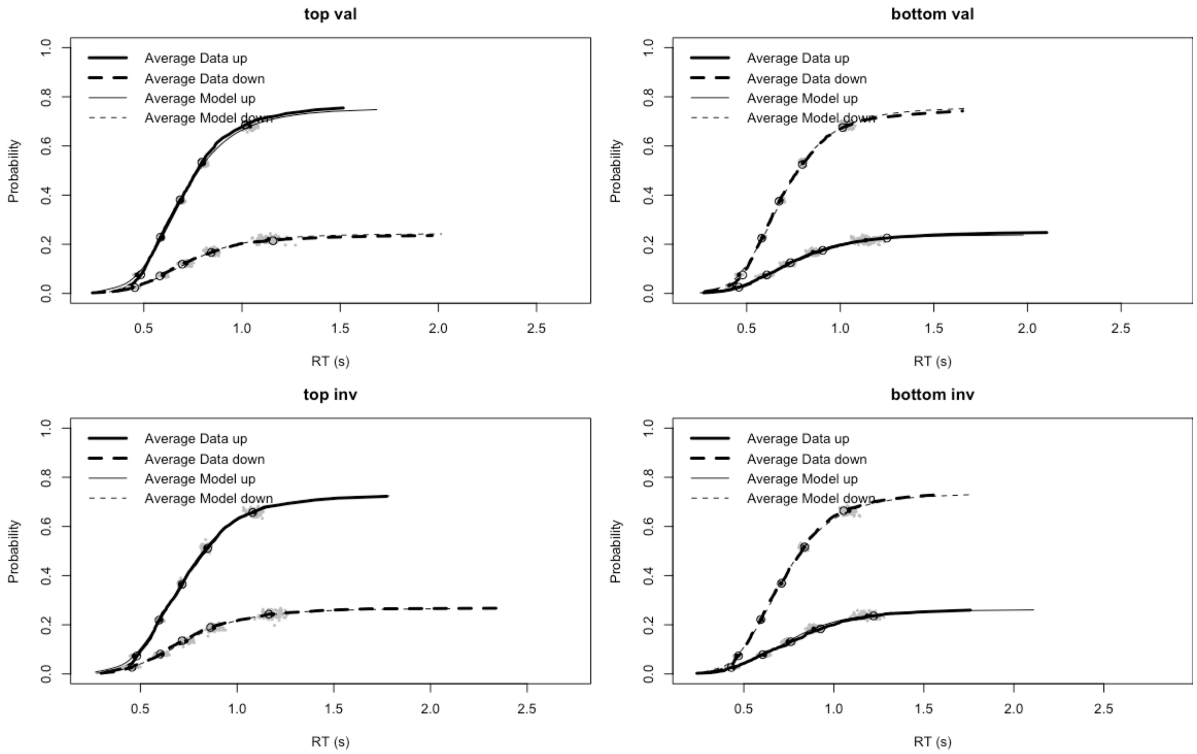

Supplementary Figure S8. Cumulative distribution functions for data (thick lines) and fits (line grey lines) of the LBA model for Experiment 1a Fixation task. Each panel contains results for both up and down responses at each level of stimulus (top and bottom) and cue validity (valid and invalid). Symbols mark the 10th, 30th, 50th, 70th and 90th percentile (solid for average fits, open for data). Grey points are 500 percentile estimates from fits for random draws from posterior samples; the grey line and black solid points are the average of these 500 fits.

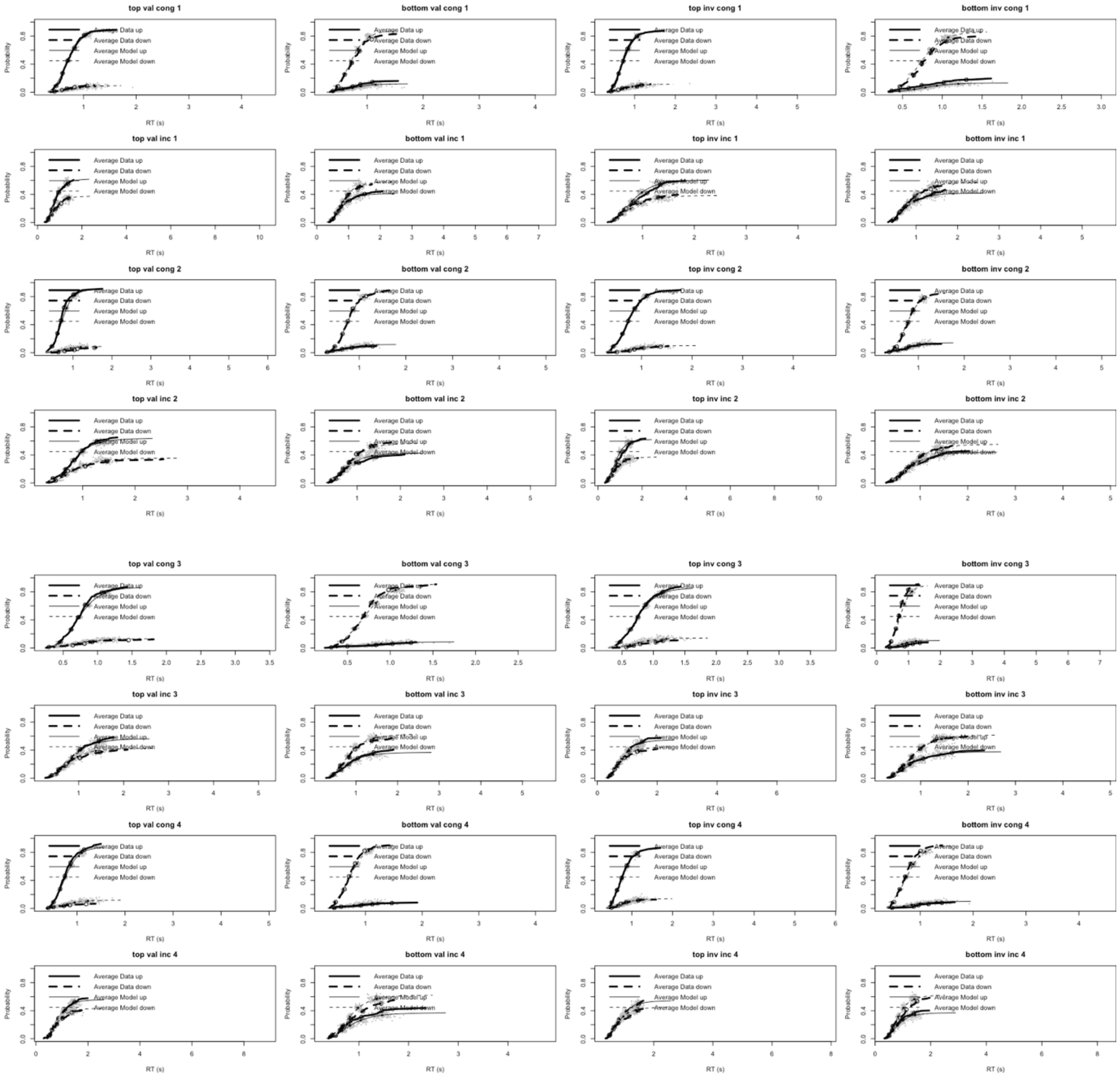

Supplementary Figure S9. Cumulative distribution functions for data (thick lines) and fits (line grey lines) of the LBA model for Experiment 1a Dual task. Each panel contains results for up and down responses at every level of stimulus (top vs. bottom), validity (valid vs. invalid), congruent (congruent vs. incongruent) and saccade instruction (1-4). Symbols mark the 10th, 30th, 50th, 70th and 90th percentile (solid for average fits, open for data). Grey points are 500 percentile estimates from fits for random draws from posterior samples; the grey line and black solid points are the average of these 500 fits.

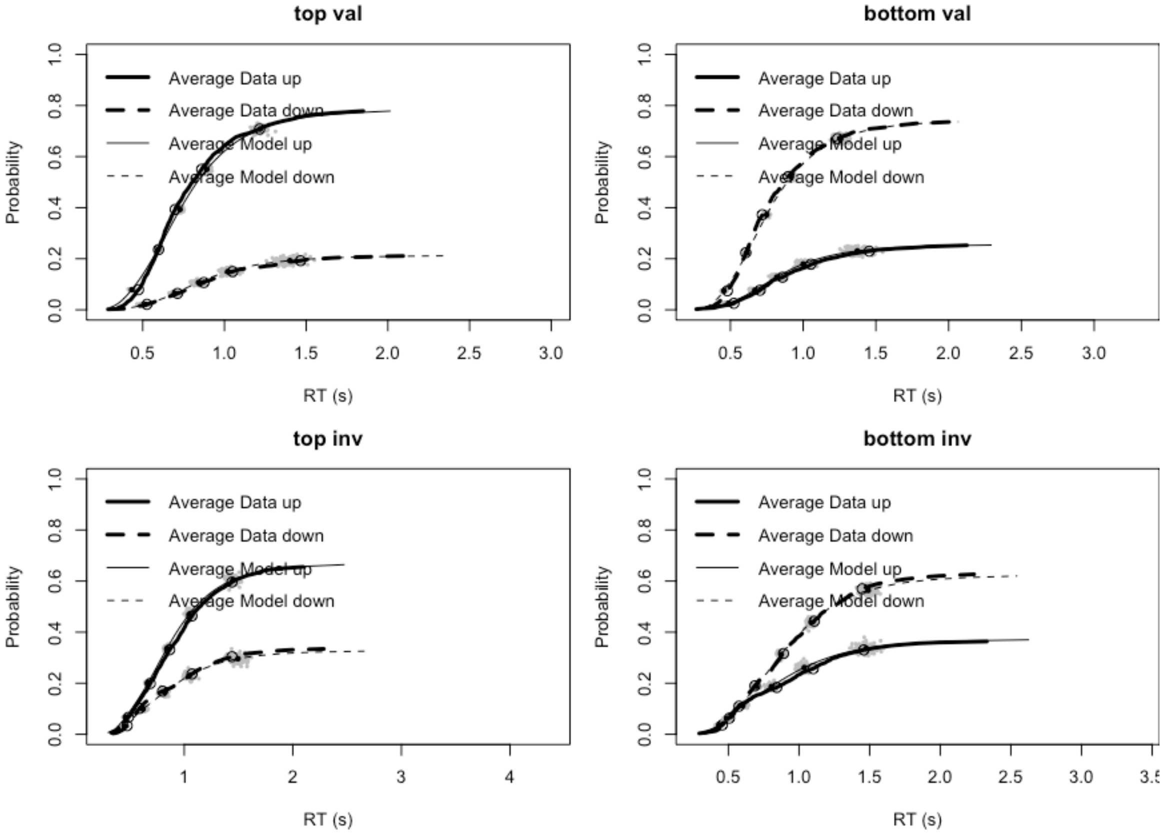

Supplementary Figure S10. Cumulative distribution functions for data (thick lines) and fits (line grey lines) of the LBA model for Experiment 1b Fixation task. Each panel contains results for both up and down responses at each level of stimulus (top and bottom) and validity (valid vs. invalid). Symbols mark the 10th, 30th, 50th, 70th and 90th percentile (solid for average fits, open for data). Grey points are 500 percentile estimates from fits for random draws from posterior samples; the grey line and black solid points are the average of these 500 fits.

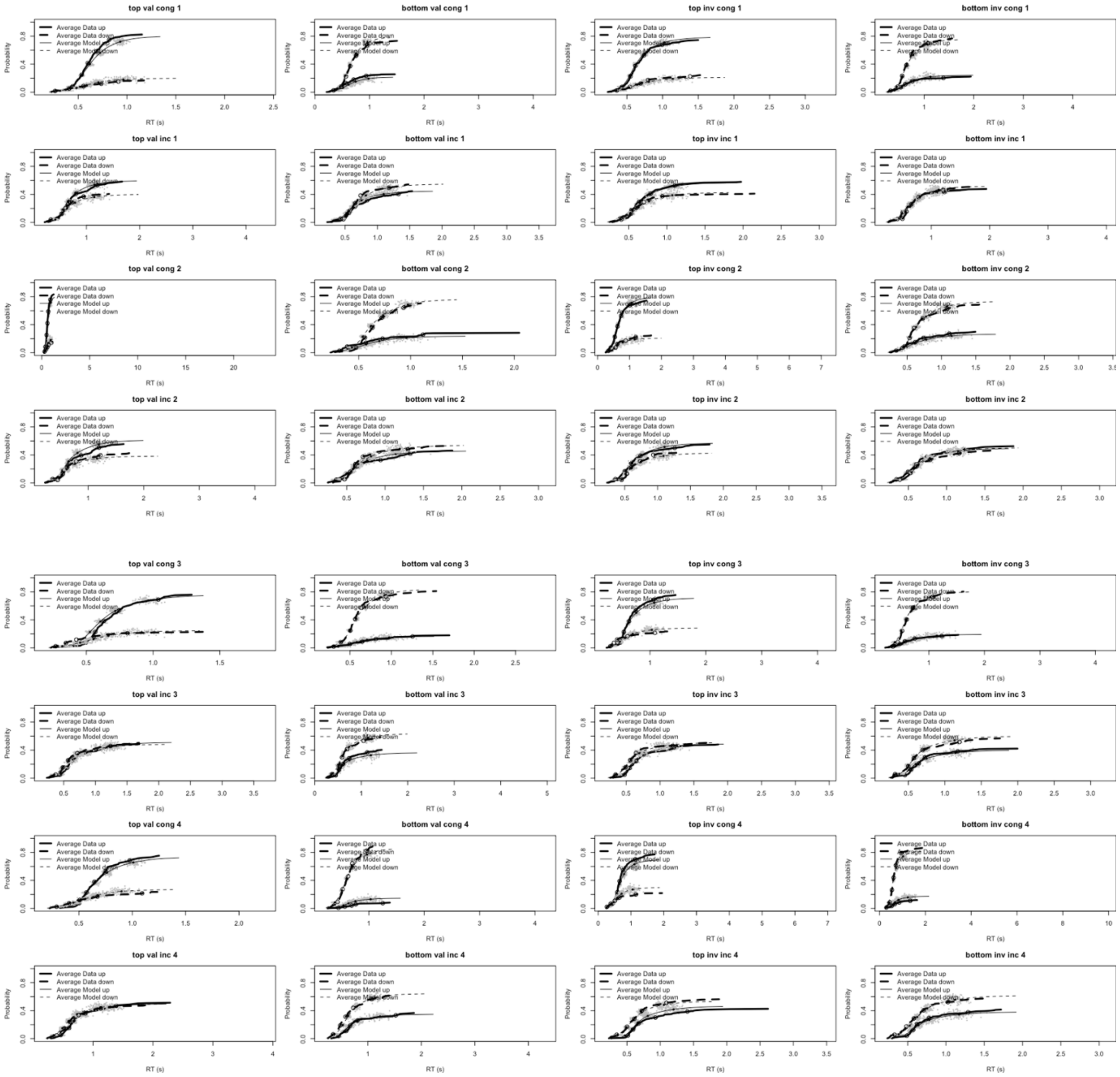

Supplementary Figure S11. Cumulative distribution functions for data (thick lines) and fits (line grey lines) of the LBA model for Experiment 1b Dual task. Each panel contains results for up and down responses at every level of stimulus (top vs. bottom), validity (valid vs. invalid), congruent (congruent vs. incongruent) and saccade instruction (1-4). Symbols mark the 10th, 30th, 50th, 70th and 90th percentile (solid for average fits, open for data). Grey points are 500 percentile estimates from fits for random draws from posterior samples; the grey line and black solid points are the average of these 500 fits.

Discussion of Evidence Accumulation Modelling Analysis: Unique arrow cueing effects.

In addition to addressing our three preregistered hypotheses we also assessed how the magnitude of the arrow cueing effect varied as a function of eye movement preparation or fixation. The results of this analysis uncovered some unexpected findings which we address below.

First, inspection of how threshold estimates varied across the fixation and dual task for Experiment 1b revealed participants to require more evidence in order to trigger a decision on the fixation version of the task relative to the dual task. This is surprising in light of a number of studies reporting increased response caution for dual relative to single task trials (Boag, Strickland, Heathcote, et al., 2019; Boag, Strickland, Loft, et al., 2019; Strickland et al., 2019; Strickland et al., 2017; Strickland et al., 2018).

Second, once these differences were accounted for the magnitude of the cueing effect was also found to vary by eye movement task. Specifically, the cue validity effect was revealed to be four times larger on fixation only trials relative to dual task trials. While this result is inconsistent with studies that have employed sudden onset peripheral cues, it is consistent with previous findings that have employed centrally presented arrows. Parker and colleagues (2020), using a different dual task design, manipulated spatial attention with a 75% valid centrally presented arrow. Eye movements, on the other hand, were instructed to made in the same direction as the arrow, opposite the arrow or not made at all upon the onset of three randomised auditory tones that differed in pitch. Inspection of the magnitude of these effects, found the cueing effect on fixation trials to similarly be four times as large that evident on eye movement trials.

We believe that the results in the present study, rather than reflecting something unique about the relationship between eye movements and spatial attention for non-social relative to social cues, instead indicate that there may be a unique relationship between

orienting mechanisms for arrow cues. Specifically. We draw this conclusion from two findings. First, from the similarities between the results for gaze cueing in the present study and the results of previous studies that have employed sudden onset peripheral cues (Parker et al., 2020a, 2021). In these tasks, spatial attention was directed with a distinctly non-social cue. Yet the pattern of dissociation across both fixation and dual tasks, as well as within the dual task itself is almost identical to that evidence on gaze cueing trials in the present study. And second, from the similarities between the results for non-predictive arrow cueing in the present study and those previously reported for centrally presented arrow cues, as discussed above.

References

Barr, D. J., Levy, R., Scheepers, C., & Tily, H. J. (2013, Apr). Random effects structure for confirmatory hypothesis testing: Keep it maximal. *J Mem Lang, 68*(3). https://doi.org/10.1016/j.jml.2012.11.001

Boag, R. J., Strickland, L., Heathcote, A., Neal, A., & Loft, S. (2019, Dec). Cognitive control and capacity for prospective memory in complex dynamic environments. *Journal of Experimental Psychology: General, 148*(12), 2181-2206. https://doi.org/10.1037/xge0000599

Boag, R. J., Strickland, L., Loft, S., & Heathcote, A. (2019). Strategic attention and decision control support prospective memory in a complex dual-task environment. *Cognition, 191*. https://doi.org/10.1016/j.cognition.2019.05.011

Brooks, S. P., & Gelman, A. (1998). General methods for monitoring convergence of iterative simulations. *Journal of Computational and Graphical Statistics, 7*(4), 434-455. https://doi.org/10.1080/10618600.1998.10474787

Bürkner, P.-C. (2017, 08/29). brms: An R Package for Bayesian Multilevel Models Using Stan. *Journal of Statistical Software, 80*(1), 1 - 28. https://doi.org/10.18637/jss.v080.i01

Gelman, A. (2006). Prior distributions for variance parameters in hierarchical models (comment on article by Browne and Draper). *Bayesian analysis, 1*(3), 515-534.

McElreath, R. (2020). *Statistical Rethinking: A Bayesian Course with Examples in R and Stan*. CRC Press.

Parker, S., Heathcote, A., & Finkbeiner, M. (2020a). Spatial Attention and Saccade Preparation Both Independently Contribute to the Discrimination of Oblique Orientations. *Adv Cogn Psychol, 16*(4), 329-343. https://doi.org/10.5709/acp-0307-8

Parker, S., Heathcote, A., & Finkbeiner, M. (2020b, Apr). Using evidence accumulation modeling to quantify the relative contributions of spatial attention and saccade preparation in perceptual tasks. *J Exp Psychol Hum Percept Perform, 46*(4), 416-433. https://doi.org/10.1037/xhp0000723

Parker, S., Heathcote, A., & Finkbeiner, M. (2021, Feb). Establishing the separable contributions of spatial attention and saccade preparation across tasks with varying acuity demands. *J Exp Psychol Hum Percept Perform, 47*(2), 172-188. https://doi.org/10.1037/xhp0000881

Strickland, L., Elliott, D., Wilson, M. D., Loft, S., Neal, A., & Heathcote, A. (2019, Apr 15). Prospective memory in the red zone: Cognitive control and capacity sharing in a complex, multi-stimulus task. *Journal of Experimental Psychology: Applied*. https://doi.org/10.1037/xap0000224

Strickland, L., Heathcote, A., Remington, R. W., & Loft, S. (2017). Accumulating evidence about what prospective memory costs actually reveal. *Journal of Experimental Psychology: Learning, Memory, and Cognition, 43*(10), 1616-1629. https://doi.org/10.1037/xlm0000400

Strickland, L., Loft, S., Remington, R. W., & Heathcote, A. (2018). Racing to remember: A theory of decision control in event-based prospective memory. *Psychological Review, 125*(6), 851-887. https://doi.org/10.1037/rev0000113
